# DeepPVC: prediction of a partial volume-corrected map for brain positron emission tomography studies via a deep convolutional neural network

**DOI:** 10.1186/s40658-022-00478-8

**Published:** 2022-07-30

**Authors:** Keisuke Matsubara, Masanobu Ibaraki, Toshibumi Kinoshita, for the Alzheimer’s Disease Neuroimaging Initiative

**Affiliations:** 1 Department of Management Science and Engineering, Faculty of System Science and Technology, Akita Prefectural University, 84-4 Aza Ebinokuchi Tsuchiya, Yurihonjo, 015-0055 Japan; 2 Department of Radiology and Nuclear Medicine, Research Institute for Brain and Blood Vessels, Akita Cerebrospinal and Cardiovascular Center, Akita, 010-0874 Japan

**Keywords:** Deep learning, Partial volume correction, PET, Amyloid

## Abstract

**Background:**

Partial volume correction with anatomical magnetic resonance (MR) images (MR-PVC) is useful for accurately quantifying tracer uptake on brain positron emission tomography (PET) images. However, MR segmentation processes for MR-PVC are time-consuming and prevent the widespread clinical use of MR-PVC. Here, we aimed to develop a deep learning model to directly predict PV-corrected maps from PET and MR images, ultimately improving the MR-PVC throughput.

**Methods:**

We used MR T1-weighted and [^11^C]PiB PET images as input data from 192 participants from the Alzheimer’s Disease Neuroimaging Initiative database. We calculated PV-corrected maps as the training target using the region-based voxel-wise PVC method. Two-dimensional U-Net model was trained and validated by sixfold cross-validation with the dataset from the 156 participants, and then tested using MR T1-weighted and [^11^C]PiB PET images from 36 participants acquired at sites other than the training dataset. We calculated the structural similarity index (SSIM) of the PV-corrected maps and intraclass correlation (ICC) of the PV-corrected standardized uptake value between the region-based voxel-wise (RBV) PVC and deepPVC as indicators for validation and testing.

**Results:**

A high SSIM (0.884 ± 0.021) and ICC (0.921 ± 0.042) were observed in the validation and test data (SSIM, 0.876 ± 0.028; ICC, 0.894 ± 0.051). The computation time required to predict a PV-corrected map for a participant (48 s without a graphics processing unit) was much shorter than that for the RBV PVC and MR segmentation processes.

**Conclusion:**

These results suggest that the deepPVC model directly predicts PV-corrected maps from MR and PET images and improves the throughput of MR-PVC by skipping the MR segmentation processes.

**Supplementary Information:**

The online version contains supplementary material available at 10.1186/s40658-022-00478-8.

## Background

Positron emission tomography (PET) has been used to quantify biological processes such as the deposition of amyloid-beta plaques [1–4] and neurofibrillary tangles [5–8] in the cerebral cortex that occur in neurodegenerative disorders, including Alzheimer’s disease (AD). The low spatial resolution of PET images, typically 5–8 mm full width at half maximum (FWHM), results in a spill-out of radioactivity concentration from regions of interest and spill-in from marginal regions; this phenomenon is referred to as the “partial volume effect” [9]. Morphological changes in the regions of interest (ROI) enhance the partial volume effect, especially if the size of the target regions decreases; for example, thinning of the cortical gyri due to brain atrophy results in a stronger spill-out from gray matter (GM) regions, thereby underestimating the cortical radioactivity concentration. This indicates the need to correct the spillover from GM for quantitative and cross-sectional studies using amyloid PET.

Several partial volume correction (PVC) methods guided by anatomical imaging, such as magnetic resonance (MR) and computed tomography imaging, have been proposed [10–17]. For example, Rousset et al. proposed the geometric transfer matrix (GTM) method, which involves the calculation of a matrix that includes spillover among ROIs drawn on an MR image for region-wise PVC [15]. Furthermore, Thomas et al. proposed extending the GTM method to voxel-wise PVC [17]. Some MR imaging-guided PVC (MR-PVC) methods have been available with software packages such as PMOD (http://www.pmod.com/web/) and FreeSurfer (https://surfer.nmr.mgh.harvard.edu/fswiki/PetSurfer). These methods are widely used in brain PET studies [18–22].

Deep learning, a machine learning method that uses a neural network comprising numerous layers [23, 24], recently became an extensively used technique for constructing artificial intelligence. Deep learning techniques have been employed for various tasks in medical imaging of the brain, such as brain tumor segmentation [25–28], automated AD detection [29–31], and stroke lesion segmentation [32–34]. Multiple research groups have proposed parcellation of the cerebral cortex with a convolutional neural network (CNN) model trained with parcellation maps acquired by FreeSurfer [35, 36]. The parcellation estimated by Henschel’s model matched well with FreeSurfer’s parcellation (89.08% in Dice coefficient) and manual segmentation (80.19%), implying that the CNN model can learn human brain anatomy and provide accurate cortical region parcellation.

We hypothesized that the CNN model would estimate PV-corrected maps from MR and PET images. To verify this hypothesis, we trained the U-shaped CNN model with skip connections (U-Net) [37] using T1-weighted MR and [^11^C]PiB PET images as inputs and a PV-corrected map as a target. We referred to the trained U-Net model as “deepPVC.” To demonstrate the importance of both anatomical and physiological information for predicting PV-corrected maps, we compared the model trained with only PET images to those trained with MR and PET images. The conventional MR-PVC is affected by error sources, such as misregistration between MR and PET images, and inaccurate point spread function (PSF). We tested the hypothesis that the effects of the PET-MR misregistration and inaccurate PSF on the deepPVC were the same as those of the conventional MR-PVC method. Furthermore, we predicted PV-corrected maps for brain [^18^F]FDG PET images using the model trained with [11C]PiB PET images to demonstrate whether the trained model learned the pure partial volume effect or uptake patterns specific to [^11^C]PiB.

## Methods

### Dataset

The data analyzed in this study were obtained from the Alzheimer’s Disease Neuroimaging Initiative (ADNI) database. ADNI primarily aimed to investigate whether a combination of measurements from serial MRI, PET, clinical and neuropsychological assessments, and other biological markers can be used to measure the progression of mild cognitive impairment (MCI) and early AD (for up-to-date information, see www.adni-info.org).

We downloaded 192 image sets of PiB PET and MR three-dimensional (3D) T1-weighted images from the ADNI database. The PET and MR images were acquired from 93 participants, including 16 healthy controls (HC), 59 patients with MCI, and 18 patients with AD. One, two, and three follow-up scans and a baseline scan were performed for 43, 25, and 2 participants, respectively. Only a baseline scan was performed for the remaining 23 participants. No participants experienced conversion from HC to MCI or AD or from MCI to AD.

For the PET input, we downloaded PET data that were preprocessed by the co-registration of each frame to the first frame and averaged the frames (5 min × four frames starting 50 min after the [^11^C]PiB injection; this is termed “Coregister, Averaged” in the ADNI database). We smoothed the downloaded PET images using a 3D Gaussian kernel to adjust the PSF for similar PET images among all ADNI sites. The smoothing kernel employed in this study was the same as that used in the “post-processed” image, named “Co-reg, Avg, Std Img, and Vox Siz, Uniform resolution” in the ADNI database. The smoothed PET images had a uniform isotropic resolution of an 8 mm FWHM.

For the MR input, we downloaded thin-sliced MR T1-weighted images from the ADNI database. The downloaded MR T1-weighted images were resampled to 256 × 256 × 256 voxels with dimensions of 1 × 1 × 1 mm^3^. The resampled MR images were analyzed using FreeSurfer (https://surfer.nmr.mgh.harvard.edu) to automatically label the volumes of interest (VOIs) [38, 39] for PVC and subsequent VOI analysis. A total of 113 labeled VOIs were identified based on the Desikan/Killiany atlas [40] and termed as “aparc + aseg” in the FreeSurfer software. To save computation time in the PVC processes, we merged the 113 VOIs into 44 regions (22 regions in each hemisphere) based on definitions from previous analysis by the ADNI PiB PET Core [41]. Details regarding the process of merging VOIs are presented in Additional file 1: Table S1. To examine spillover to non-brain tissues and air in the PVC, we added a VOI comprising a 15 mm “shell” surrounding the outer surface of the brain. The VOI map for a representative case is shown in Additional file 1: Fig. S1. To avoid memory errors during training, MR images were down-sampled to 128 × 128 × 128 voxels with 2 × 2 × 2 mm^3^ before registration of PET images and PVC processes as follows.

We considered maps corrected for the partial volume effect based on the region-based voxel-wise (RBV) method [17] as the target images for training. RBV PVC is a voxel-wise extension for the GTM method. The PVC-optimized registration (PoR) framework [42] was applied to compensate for the misregistration between the MR and PET images. Briefly, the PoR framework iteratively performed PVC and registration between the smoothed PV-corrected map and uncorrected PET image. Next, the final PV-corrected map was generated by performing RBV PVC using a misregistration-compensated PET image.

We considered the 156 image sets of MR and PET images as the training data. Thirty-six image sets acquired from sites other than the training datasets were regarded as test dataset.

### Training

**Fig. 1:**
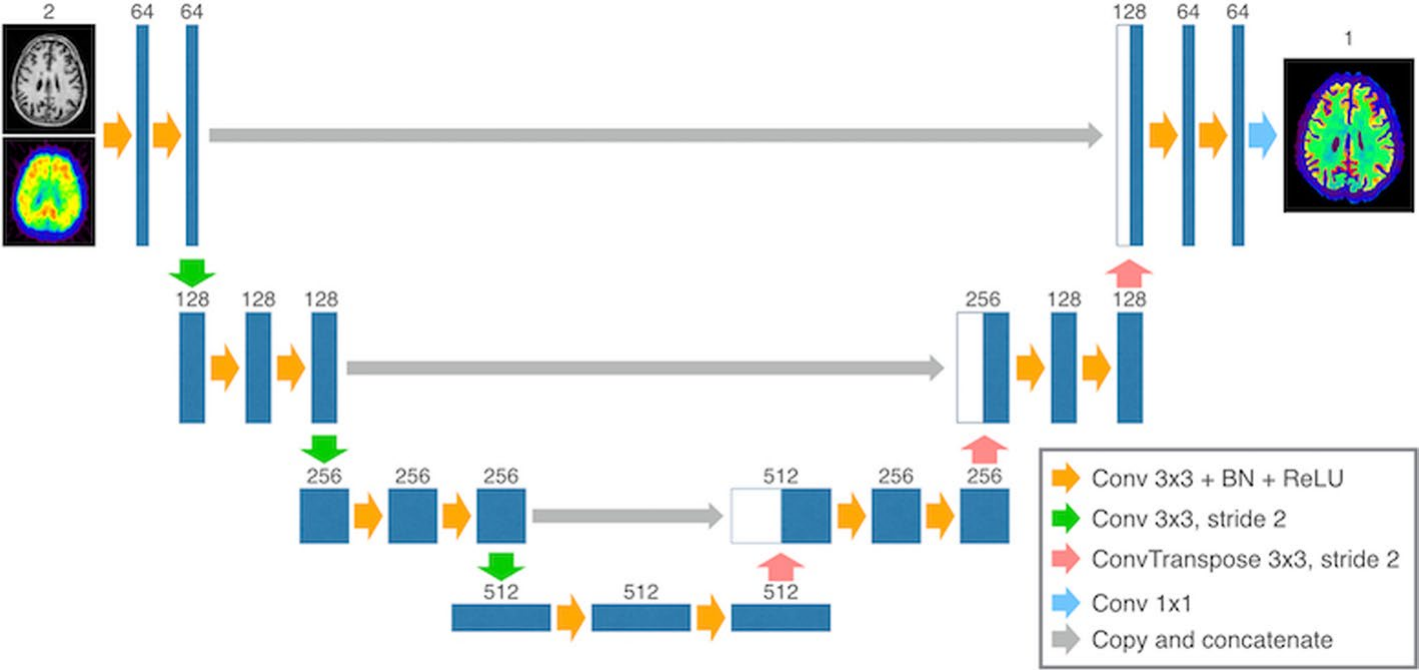
Convolutional neural network model used in this study. The numbers on each data layer indicate the number of channels

The 2D U-Net [37] used in this study is shown in Fig. 1. The 2D U-Net was trained slice-by-slice using the training dataset containing 156 image sets. In brief, the U-Net contains the encoder and decoder parts. The encoder compresses the data to extract the robust image features, while the decoder part mirrors the encoder’s structure and restores a desirable image from the extracted features. Each level of the encoder and decoder parts contains two convolutional layer blocks. Each block includes a convolutional layer, a batch normalization layer to prevent internal covariance shifts [43], and an activation layer with rectified linear units [44]. Down- and up-sampling were performed of the encoder and decoder parts, with convolutional and transposed convolutional layers with a stride of two. The number of channels for data was doubled in the down-sampling and reduced by half in the up-sampling. We empirically set the number of down- and up-samplings to three. Skip connections at each level of the network were added to prevent the loss of spatial information. Finally, the output images were recovered from the final image features using a convolutional layer with a 1 × 1 kernel. The total number of parameters for the U-Net was 8.56 million. The orientation of the input and output slices was axial.

Further, we trained the network weights by minimizing the mean squared error between the real and predicted output images. The weights were optimized using the Adam method [45]. The hyperparameters for Adam, *β*_1_ and *β*_2_, were set to 0.723 and 0.999, respectively. The update of the weights was implemented in batches, including 16 image sets, and iterated with 400 epochs. The initial learning rate was set to 0.0018 and linearly decayed from the 200th epoch to the end of the learning process. The final learning rate in the end was zero. The *β*_1_, batch size, number of epochs, and initial learning rate were optimized by Bayesian optimization using the Optuna library (https://optuna.org/) [46]. Data augmentation was performed of the training data with rotation by angle randomly selected in range between − 30 and 30 degrees, and horizontal flipping. The training was implemented using the PyTorch library (https://pytorch.org/) [47].

Blank slices not showing head and brain on the input and output images were omitted for efficient training and prediction by the U-Net. The intensities on input PET and MR images were standardized by dividing with the average of each individual image. The output PV-corrected maps were standardized by dividing with the average of the individual PET image as input.

### Validation

To validate whether the trained deepPVC model learns features for PVC from the MR and PET images, we performed training using only the PET images as well as using the PET and MR images and named the groups “deepPVC_PET_” and “deepPVC_MRI+PET_,” respectively. Model performance was subjected to sixfold cross-validation. The 156 image sets were split into six data subsets—five as training data and one as validation data—and trained and evaluated the trained model six times to validate all subsets. The data were split with avoiding duplication of subject between training and validation subsets. We compared the following metrics between the two deepPVC models: (1) the structural similarity index (SSIM) [48] between the real and predicted PV-corrected maps; and (2) regional standardized uptake values (SUVs) in the VOIs on the real and predicted PV-corrected maps. The SSIM assesses the structural and perceptual similarities between the two images. The SSIM was calculated using the scikit-image library (https://scikit-image.org/) [49].

The regional SUVs on the VOIs were compared to assess the quantitative correspondence between the real and predicted PV-corrected maps. The intraclass correlation coefficient for absolute agreement of a single measure (ICC[2,1]) between the real and predicted PV-corrected SUVs for each individual was calculated as an index for the quantitative correspondence of PV-corrected SUVs. The ICC was calculated using the pingouin library (https://pingouin-stats.org/) [50]. To demonstrate the voxel-level correspondence between the real and predicted PV-corrected maps, we constructed two-dimensional (2D) histograms between the real and predicted PV-corrected SUVs on voxels in the brain for each individual.

Differences in the SSIM and ICC among the trained models were tested using a pairwise *t* test with correction for multiple comparisons using Bonferroni’s method. The SSIM and ICC of the uncorrected and real PV-corrected PET images were used as references to compare the models and test for differences between them.

### Test with [^11^C]PiB PET data

The trained deepPVC model was tested against the test data: 36 image sets acquired at different sites from those of the training data to assess the trained model performance upon generalization. Note that the PET scanner used for the test dataset differed from that used for the training/validation dataset, while the MR scanners were the same for both datasets. The lists of PET and MR scanners are presented in Additional file 1: Table S2. We tested the model trained with all 156 image sets of the training/validation dataset. The SSIM and ICC for the predicted PV-corrected maps were calculated for the test data, as for the validation data. The differences in the SSIM and ICC between the validation and test data were tested using Welch’s *t* test.

The computer used in the test had an Intel Xeon E5-1650 v3 3.50 GHz central processing unit (6 cores and 12 threads), four graphics processing units (GPUs), GeForce GTX TITAN X 12 GB, and eight 8-GB memory cards (total, 64 GB). We measured the computation time with versus without the GPU; for reference, the computation time required to perform RBV PVC was also measured.

### Test with over-smoothed PET images

To demonstrate the effect of PSF inaccuracy on deepPVC and whether the trained deepPVC model learned PSF information, we tested the model on excessively smoothed PET images. We hypothesized that, if the trained model learned the PSF information, the PSF mismatch between the PSF true and assumed in the training would affect the predicted PV-corrected maps as with conventional MR-PVC. We excessively smoothed the PET images for the test data using 6.0 and 8.9 mm FWHM Gaussian kernels, resulting in a final resolution of 10 and 12 mm FWHM, respectively. We calculated the differences between the PV-corrected SUVs predicted for the original and over-smoothed PET images using the trained deepPVC_MRI+PET_ model. For reference, we also performed RBV PVC for the smoothed PET images and compared the differences in the PV-corrected SUV with the deepPVC.

### Test with misaligned PET images

To demonstrate the effect of misregistration between the PET and MR images on deepPVC, we arbitrarily realigned the PET images for the test dataset. We then predicted the PV-corrected maps using deepPVC with the arbitrarily realigned PET and the original MR images as input data. The realignment in a single direction, shift, or rotation on the x-, y-, or z-axis was performed by ± 4, 8, or 12 mm, or ± 4, 8, or 12 degrees, respectively. We calculated the differences in regional PV-corrected SUVs from those without realignment. To compare the robustness of the misregistration between conventional PVC and deepPVC, we also performed RBV PVC for the realigned PET images.

### Test with PET images acquired with a radiotracer other than [^11^C]PiB

To determine whether the trained model learned uptake patterns specific to [^11^C]PiB, we tested the trained model on the acquired data using a tracer other than [^11^C]PiB. We assumed that the trained model could successfully predict a PV-corrected map for the other tracer if the trained model learned the pure partial volume effect on the PET images. Subsequently, [^18^F]FDG PET and MR T1 images from 16 participants were downloaded from the ADNI database, including three HCs, 10 with MCI, and three with AD. Preprocessing of these MR images was performed using FreeSurfer as for the [^11^C]PiB data; co-registration between PET and MR images using the PoR method as well as the RBV PVC method was performed, as for [^11^C]PiB data. Prediction of the PV-corrected map for the [^18^F]FDG PET data and comparisons of the real and predicted maps were implemented in the same manner as the test for [^11^C]PiB.

## Results

### Validation of the deepPVC models

**Fig. 2:**
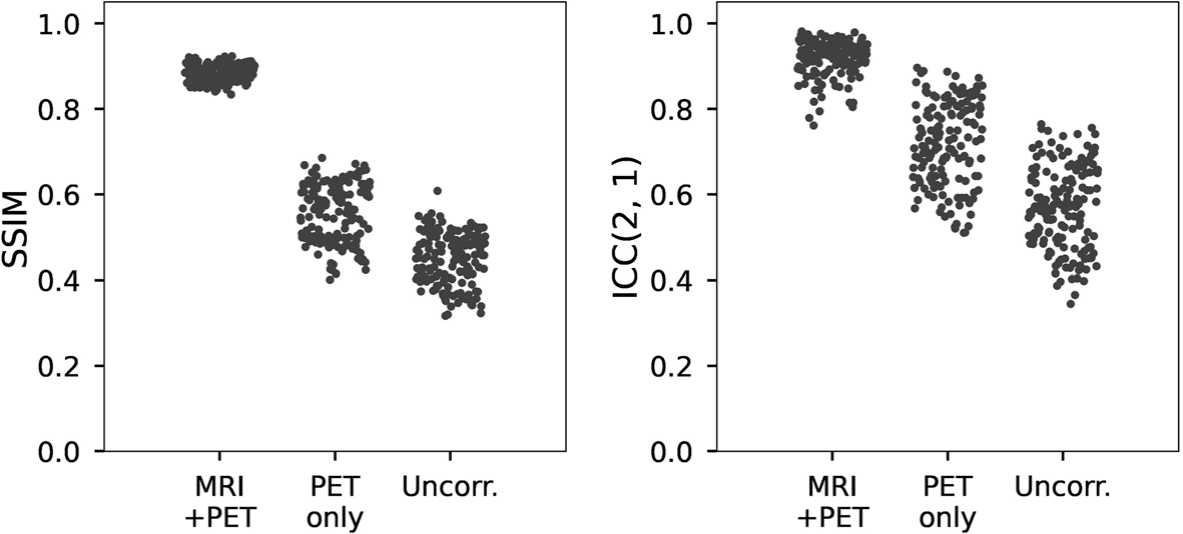
Plots for individual SSIM (left) and ICC(2,1) (right) between the real and predicted PV-corrected SUV maps for the cross-validation datasets. Please note that each dot represents an individual data point pooled from the six cross-validation datasets. ICC, intraclass correlation coefficient; SSIM, structural similarity index; SUV, standardized uptake value

**Fig. 3:**
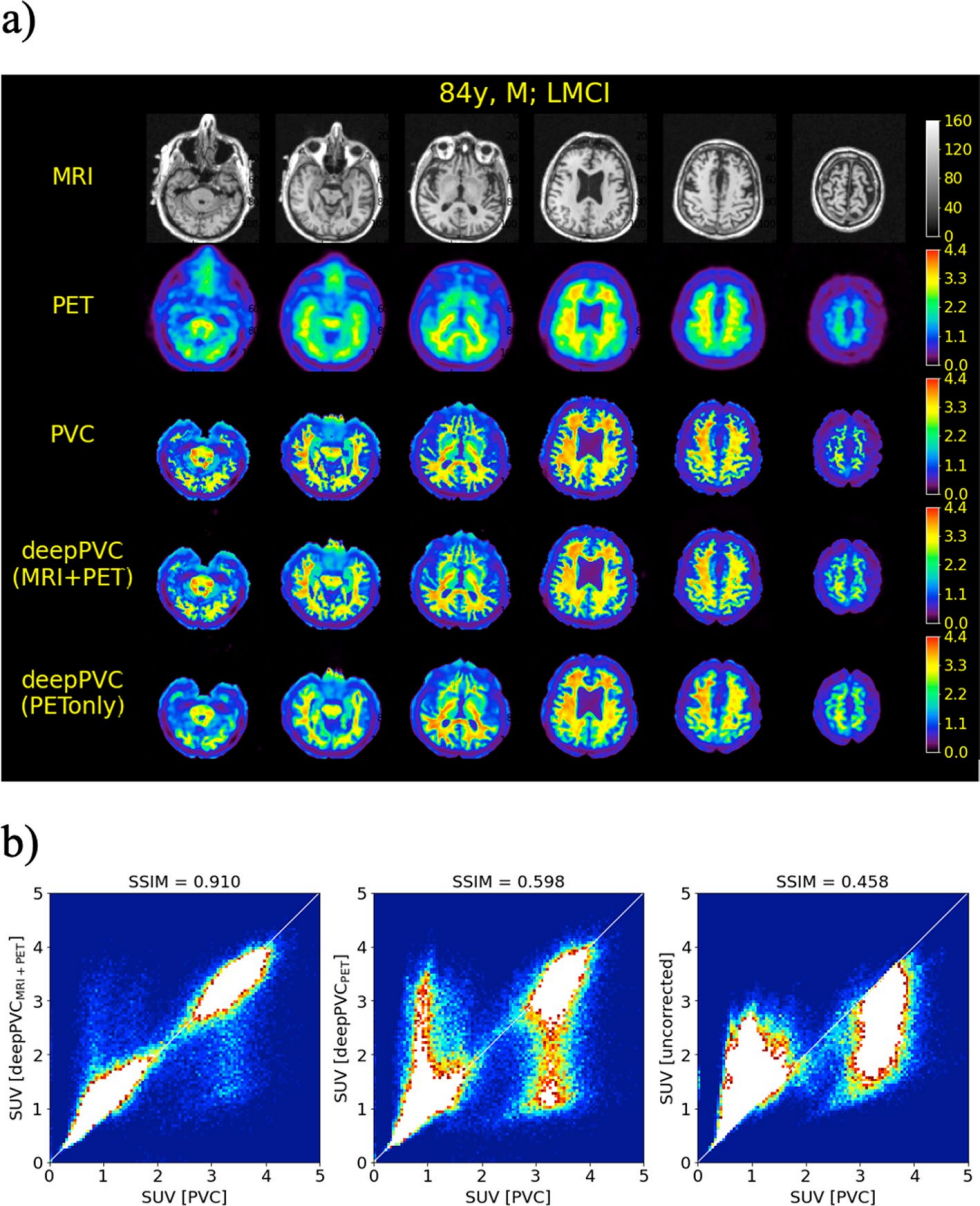
MR images, SUV maps **a**, and 2D histograms of the PV-corrected map (**b**) for the representative PiB-negative case (84 years old; male; MCI). The 2D histograms, left to right, represent maps predicted with deepPVC_MRI+PET_, deepPVC_PET_, and uncorrected PET, respectively. The white lines on the histograms indicate perfect correspondence with the real PV-corrected SUV. MCI, mild cognitive impairment; MR, magnetic resonance; MRI, MR imaging; PET, positron emission tomography; PVC, partial volume correction; SUV, standardized uptake value

**Fig. 4:**
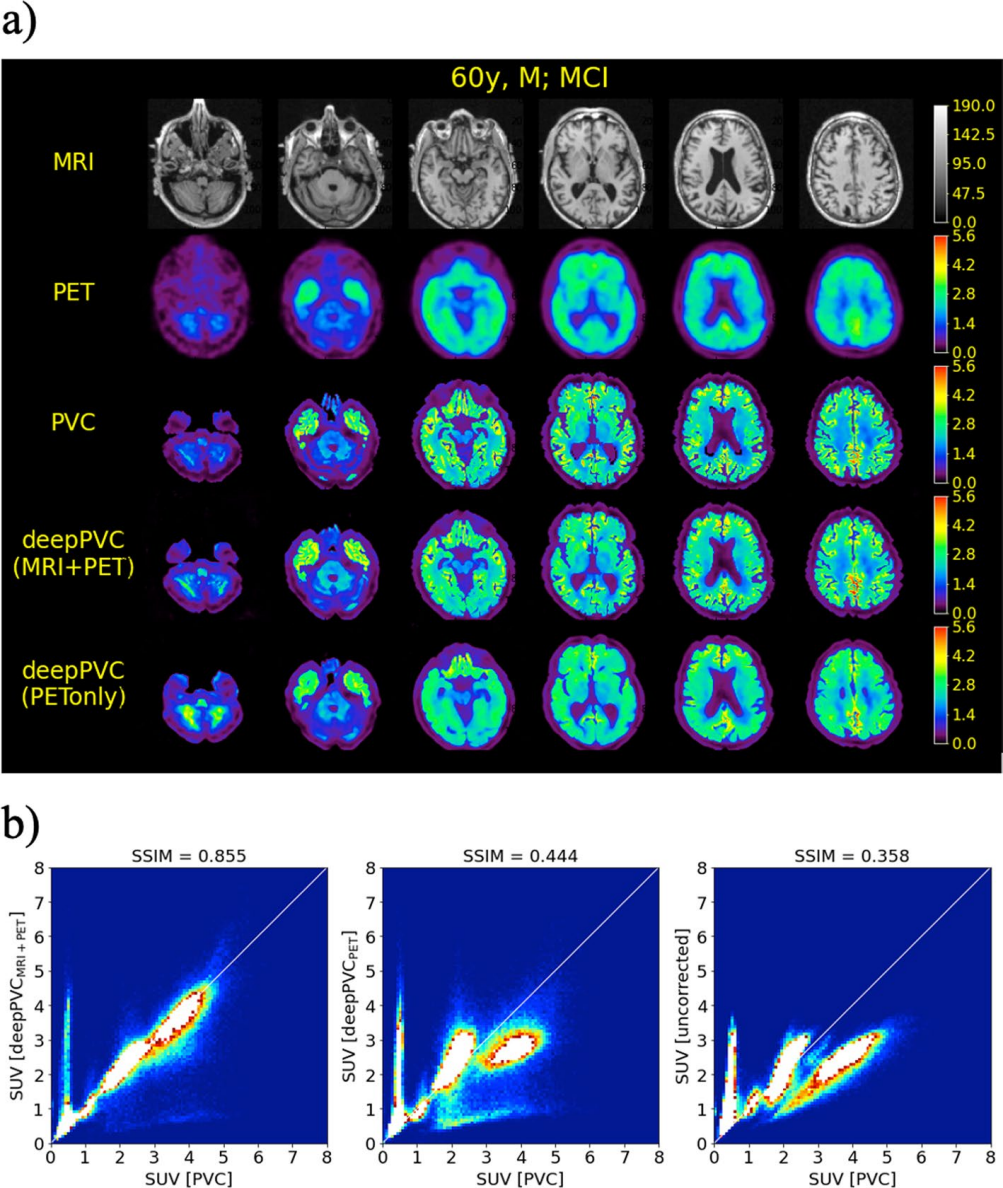
MR images, SUV maps (**a**), and 2D histograms of the PV-corrected map (**b**) for the representative case of PiB-positive (60 years old; male; MCI). The 2D histograms, left to right, represent maps predicted with deepPVC_MRI+PET_, deepPVC_PET_, and uncorrected PET, respectively. The white lines on the histograms indicate perfect correspondence with the real PV-corrected SUV. MCI, mild cognitive impairment; MR, magnetic resonance; PVC, partial volume correction; SUV, standardized uptake value

The highest structural similarity (SSIM, 0.884 ± 0.020) to the real PV-corrected maps was observed in the PV-corrected SUV maps predicted by deepPVC_MRI+PET_ (Fig. 2, Table 1). Significantly higher SSIM values were observed in all predicted SUV maps than in uncorrected PET images (*p* < 0.001). The SSIM of the PV-corrected SUV maps predicted by deepPVC_PET_ to the real maps (0.556 ± 0.069) was also significantly greater than that of the uncorrected PET images (0.450 ± 0.060). The lowest (0.020) and highest (0.069) standard deviations in SSIM were observed using deepPVC_MRI+PET_ and deepPVC_PET_, respectively. The PV-corrected maps predicted using deepPVC_MRI+PET_ were structurally more similar to the real PV-corrected maps than the maps predicted using deepPVC_PET_ (Figs. 3a, 4a). A blurred structure was observed in the maps predicted using the deepPVC_PET_. Similar trends were observed in cases other than those shown in Figs. 3a and 4a. Zoomed images for Figs. 3a and 4a are shown in Additional file 1: Fig. S2.

**Table 1:** Comparison of SSIM and ICC(2,1) among the deepPVC models for 156 subjects pooled from the six cross-validation datasets

	DeepPVC_MRI+PET_	DeepPVC_PET_	Uncorrected PET
SSIM	0.884 ± 0.020	0.556 ± 0.069	0.450 ± 0.060
ICC(2,1)	0.921 ± 0.042	0.720 ± 0.098	0.569 ± 0.097

Each value indicates the mean ± standard deviation of the 156 subjects pooled from the six cross-validation datasets.

*ICC*, intraclass correlation coefficient; *SSIM*, structural similarity index

The highest quantitative correspondence to the real PV-corrected SUV (ICC[2,1]: 0.921 ± 0.042) was observed in the PV-corrected SUV predicted using deepPVC_MRI+PET_ (Fig. 2, Table 1). The standard deviation of the ICC for deepPVC_MRI+PET_ (0.042) was much lower than that predicted by deepPVC_PET_ (0.098) and the uncorrected SUV (0.097).

Moreover, the 2D histograms for deepPVC_MRI+PET_ were nearest to the identity lines (Figs. 3b, 4b). Over- and underestimation of the PV-corrected SUV were observed, even in the histogram for deepPVC_MRI+PET_. For example, overestimation of the PV-corrected SUV was observed in low real SUV bins (approximately 0–1) in the histogram in Fig. 3b. These bins corresponded to the voxels of the cerebrospinal fluid and outside the brain. The underestimation of the PV-corrected SUV in bins with a real SUV near 2 on the histogram for deepPVC_MRI+PET_ is shown in Fig. 4b and corresponds to the voxels in various regions around the whole brain. Similar trends were observed on 2D histograms other than the cases shown in Figs. 3b and 4b.

We employed the deepPVC_MRI+PET_ model for the tests described below because it showed the best SSIM and ICC.

### Test with [^11^C]PiB PET data

**Fig. 5:**
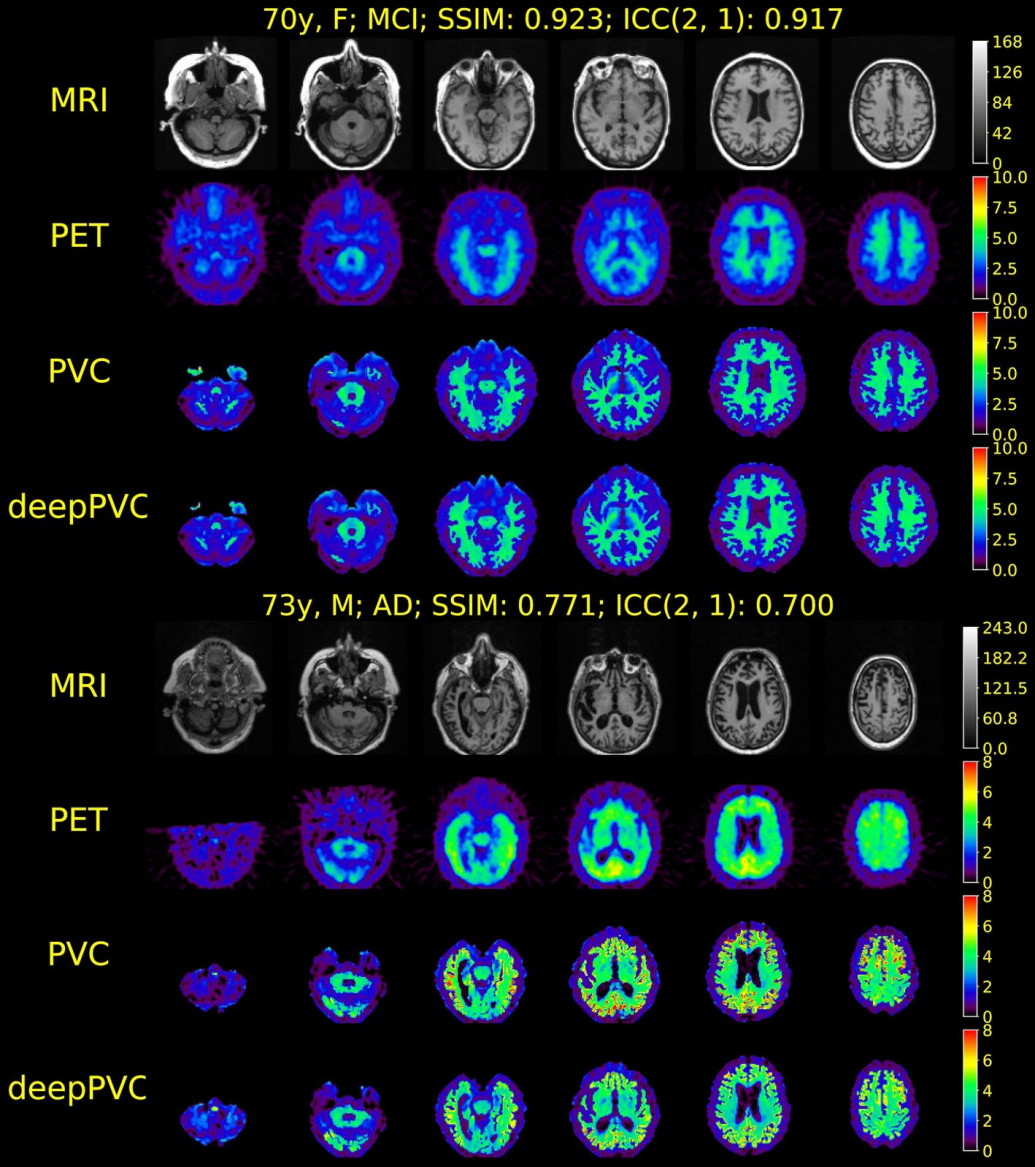
MR, PET, and real and predicted PV-corrected maps for cases with the best (top) and worst (bottom) SSIM among the [^11^C] PiB test data. MR, magnetic resonance; PET, positron emission tomography; PV, partial volume; SSIM, structural similarity index

**Fig. 6:**
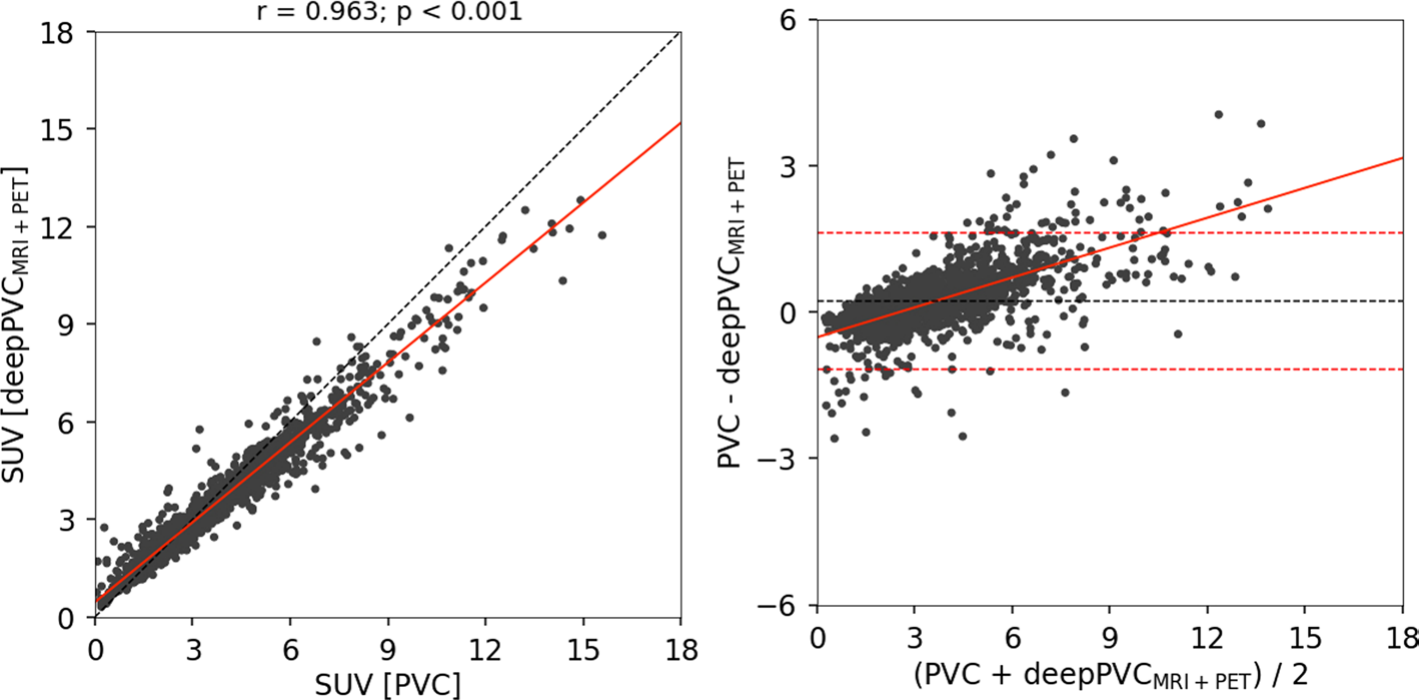
Scatter plot (left) and Bland–Altman plot (right) between the real and predicted PV-corrected SUV for the test data. Each dot indicates the regional SUV for one VOI for one subject. The dashed line indicates perfect correspondence between the real and predicted SUVs. The red line indicates a regression line. PV, partial volume; SUV, standardized uptake value; VOIs, volumes of interest

High SSIM (0.876 ± 0.028) and ICC (0.894 ± 0.051) values were obtained from the test with [^11^C]PiB data by deepPVC_MRI+PET_; however, the ICC of the test data was significantly lower than that of the cross-validation data (*p* = 0.010). The structure and uptake of the predicted maps were visually similar to those of the real PV-corrected maps in cases with a high SSIM and ICC (Fig. 5, top), while a considerable underestimation was observed in the higher PV-corrected SUV (Fig. 6). In cases with a low SSIM and ICC, differences in uptake were observed between the real and predicted PV-corrected maps (Fig. 5, bottom); overestimation in the low PV-corrected SUV is also shown in Fig. 6b. Scatter and Bland–Altman plots for each VOI are shown in Additional file 1: Fig. S3.

The computation time for training the model was 6 h 53 m. The times to predict the PV-corrected map using trained deepPVC were 8 s with GPUs and 48 s without GPUs (126 ms/slice with GPUs and 756 ms/slice without GPUs). The computation time of deepPVC without GPUs was shorter than that of RBV PVC at 1 min, 50 s.

### Test with over-smoothed PET images

**Fig. 7:**
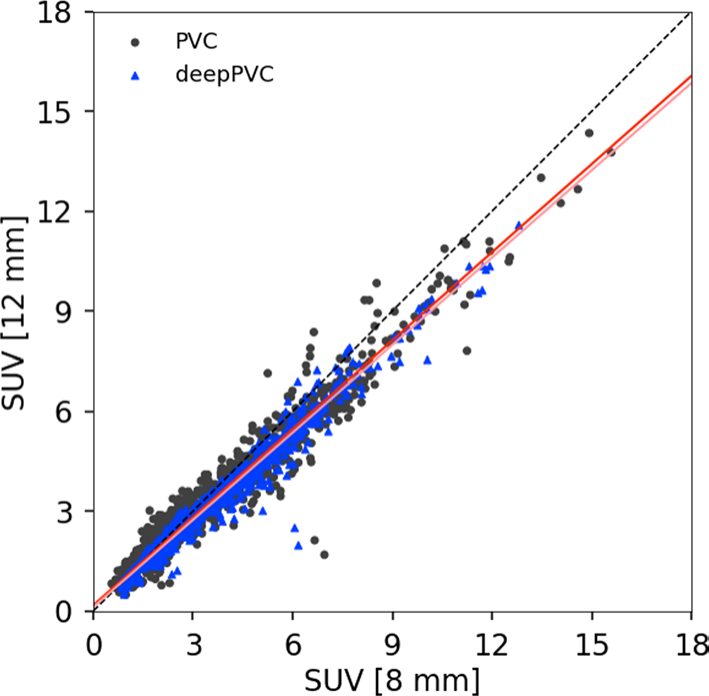
Scatter plots of SUVs PV corrected with PET images, which had final resolutions of 8–12 mm FWHM by RBV PVC (gray circle) and deep PVC (blue triangle). Regression lines for RBV PVC and deepPVC are indicated with red and pink lines, respectively. FWHM, full width half maximum; PV, partial volume; PVC, partial volume correction; RBV, region-based voxel-wise; SUV, standardized uptake value

Scatter plots of PV-corrected SUVs with PET images, which had a final resolution of 8–12 mm FWHM by RBV PVC and deep PVC, are shown in Fig. 7. Underestimation in the PV-corrected SUV for 12 mm FWHM was observed for RBV PVC and deepPVC. Regression lines were very similar between the RBV PVC (*y* = 0.883 × *x* + 0.175) and deepPVC (*y* = 0.874 × *x* + 0.121).

### Test with misaligned PET images

**Fig. 8:**
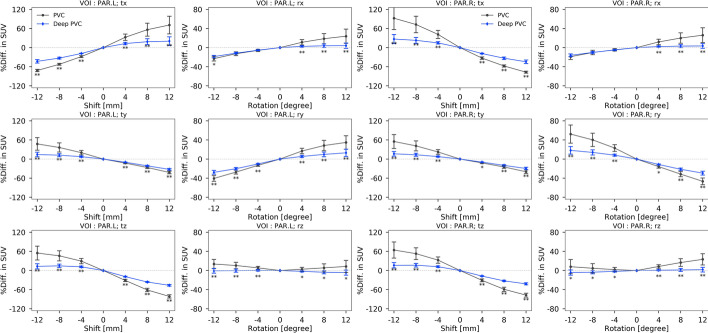
Trends of the percentage differences in PV-corrected SUV on left (first and second columns) and right (third and fourth columns) parietal cortices in response to the shifts and rotations for RBV PVC and deepPVC. Asterisks indicate significant differences between RBV PVC and deepPVC (paired *t* test; *p < 0.05; **p < 0.001). PV, partial volume; PVC, partial volume correction; RBV, region-based voxel-wise; SUV, standardized uptake value

We observed trends of significantly lower or equal percentage differences in PV-corrected SUV for deepPVC versus RBV PVC (Fig. 8 and Additional file 1: Fig. S4). Significantly higher percentage differences were observed for deepPVC versus RBV PVC in some regions and directions: 24/132 directions (132 directions = 22 regions × 6 directions/region).

### Test with [^18^F]FDG PET data

**Fig. 9:**
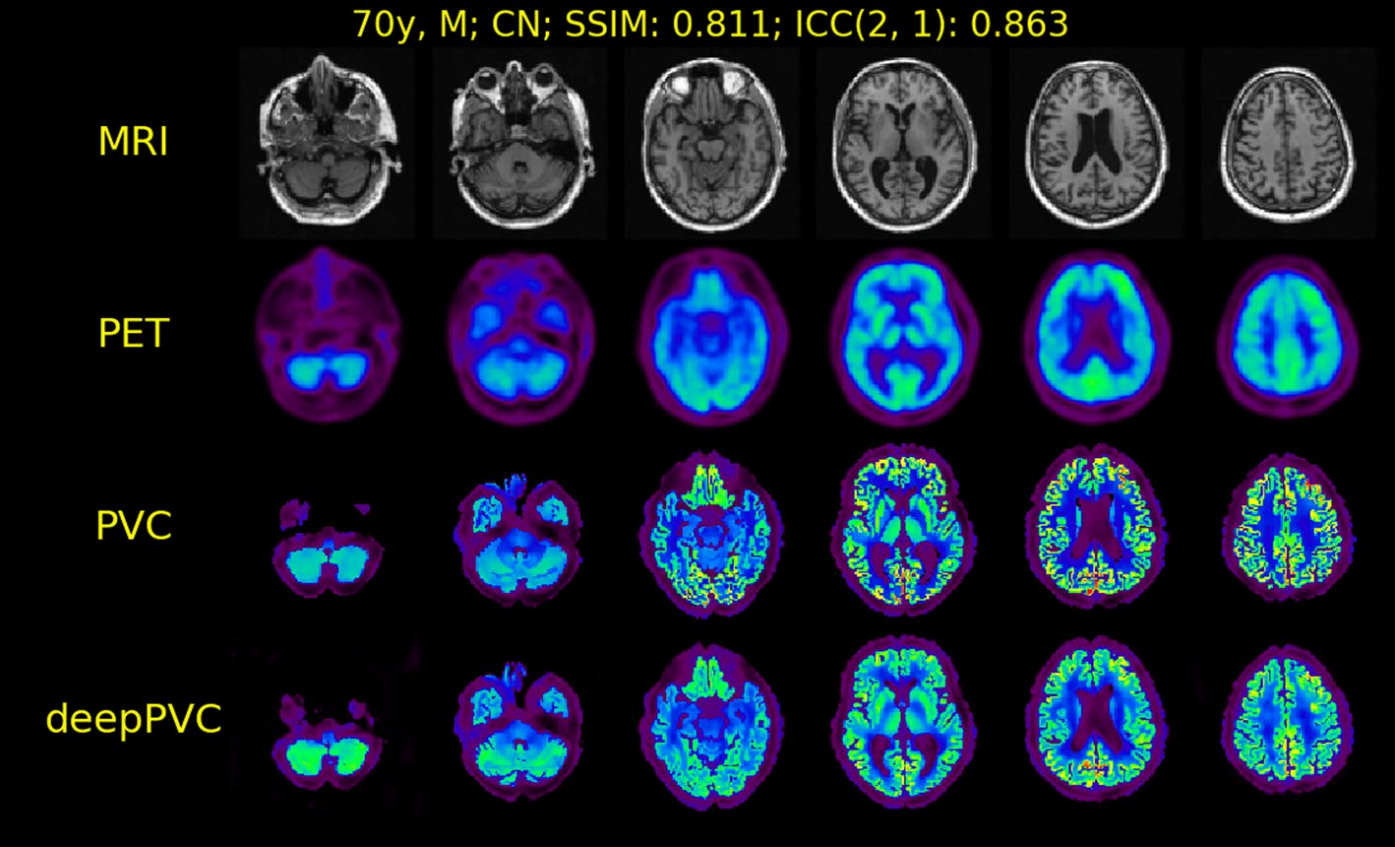
MR, PET, and real and predicted PV-corrected maps for cases with the best ICC among the [^18^F]FDG test data. CN, cognitively normal; MR, magnetic resonance; PET, positron emission tomography; PV, partial volume

Significantly lower SSIM and ICC values were observed in the test with [^18^F]FDG PET data versus [^11^C]PiB PET data (Table 2). Uptake values in the predicted PV-corrected maps were lower than those in the real PV-corrected maps and similar to those in the uncorrected PET images (Fig. 9). Similar trends were observed in other cases.

**Table 2:** SSIM and ICC(2,1) of [^11^C]PiB versus [^18^F]FDG data

	[^11^C]PiB	[^18^F]FDG	*t* values	*p* values
SSIM	0.876 ± 0.028	0.782 ± 0.040	8.452	< 0.001
ICC(2,1)	0.894 ± 0.051	0.794 ± 0.059	5.858	< 0.001

Both results are for the prediction with deepPVC_MRI+PET_.

*ICC*, intraclass correlation coefficient; *SSIM*, structural similarity index

## Discussion

We hypothesized that the deep CNN model could learn features that allow it to predict PV-corrected maps, including anatomical information of the individual brain, physiological information of the tracer uptake, and PSF on PET. The much higher SSIM and ICC observed with deepPVC_MRI+PET_ than those with deepPVC_PET_ imply that the deepPVC model learned the anatomical information from MR images as well as the physiological information from PET images. These findings are supported by previous studies that employed U-Net for MR segmentation [35, 36] and suggest that the deepPVC model implicitly learns anatomical information to perform brain segmentation.

Moreover, the much lower variability in SSIM and ICC observed with deepPVC_MRI+PET_ versus deepPVC_PET_ use implies that features from both MR and PET images are necessary for a stable prediction of PV-corrected maps using the deepPVC model. Because of the high stability of the prediction and the high correspondence between the predicted and RBV PVC maps, the deepPVC_MRI+PET_ model trained with the MR and PET images was used for the tests in this study.

The underestimation in the PV-corrected SUV for deepPVC by excessive smoothing of the input PET images can reflect mismatches between each learned and actual PSF of the input PET images. These results are consistent with those of a previous report that demonstrated the effect of PSF errors on PV-corrected SUVs [51]. Similar trends in the changes of PV-corrected SUV between deepPVC and RBV PVC imply that the deepPVC model learned information for PSF from PET images. These findings also support the hypothesis that the deepPVC model learns the features required for MR-PVC and, thus, can predict PV-corrected maps from the MR and PET images.

The high SSIM and ICC in the test data acquired using other PET scanners from sites other than the training/validation datasets suggest that the trained deepPVC model could be successfully generalized for PET scanners. However, the PET scanners used for the dataset in this study were old-generation models. Further studies are required to demonstrate the applicability of the deepPVC model to more recent PET scanners, such as scanners with time-of-flight and silicon photomultiplier detectors [52, 53].

The computation time for predicting an individual PV-corrected map in this study (48 s without GPU) was shorter than the time required to perform RBV PVC (1 min 50 s), and the total computation time of MR-PVC with MR segmentation processes, which was 4–8 h using FreeSurfer. The computation time in this study was similar to those previously reported for volumetric segmentation using the U-Net and the proposed pipeline (1 min) [35]. These results suggest that the deepPVC models improve the throughput of MR-PVC by shortening the time it takes to perform PVC and by skipping the MR segmentation processes.

The lower SSIM and ICC in the test with [^18^F]FDG PET versus [^11^C]PiB PET data implies that the deepPVC model learned tracer-specific features from the [^11^C]PiB PET images, not merely features of the partial volume effect. These results suggest the need to train the deepPVC model with PET images for the target tracer. The construction of a deepPVC model for multiple tracers, by training on PET images acquired using multiple tracers, is an alternative consideration.

Considerable underestimation of PV-corrected SUV with deepPVC reflects insufficient correction for spill-out from the target region, whereas overestimation of PV-corrected SUV in low real SUV reflects insufficient correction for spill-in from surrounding regions. These results suggest that the recovery of radioactivity with deepPVC is not as perfect as that achieved with PVC; thus, the quantitative accuracy of the predicted PV-corrected maps with deepPVC remains inferior to that of maps corrected by RBV PVC. The lower differences in PV-corrected SUV for deepPVC than RBV PVC in the test for misaligned PET images were due to insufficient recovery. Underestimation in PV-corrected SUV may be observed in case of combination of the misalignment on a single direction, as actual application. We suppose that slice-by-slice training and prediction with 2D U-Net resulted in the quantitative inaccuracy of the PV-corrected maps because a partial volume effect occurs on PET images in 3D space. However, the computational cost for the training and prediction of volume data using a 3D CNN is extremely high. Actually, we cannot optimize hyperparameters for 3D U-Net because training of the 3D U-Net with 400 epochs spends approximately 7 days with our GPU workstation. Overfitting due to the small data size of the training data in this study can be the other reason for the underestimation of PV-corrected SUV. Further studies are required to predict PV-corrected maps using a 3D CNN in a high-specification computation environment and larger training dataset.

The deepPVC cannot avoid some error sources in maps PV-corrected by MR-PVC as a training target. For example, segmentation errors in MR segmentation processes can propagate from the PV-corrected maps used as training targets to the maps predicted using the trained deepPVC model. Other error sources, such as patient motion and attenuation–emission mismatches, can also propagate from the training target to the maps predicted using the deepPVC model. To avoid misregistration between PET and MR images, we applied the PoR framework to compensate for misregistration errors in the calculation of PV-corrected maps used as training targets. Much attention should be given to the quality control of PV-corrected maps used as training targets for the deepPVC model. One possible solution to make the deepPVC model robust for the error sources is adding these errors in data augmentation on the training. For example, shifting and rotating either PET or MR images in data augmentation can make the trained model robust for the misalignment error between PET and MR images.

We applied U-Net for generating partial volume-corrected maps in this study because the U-Net is the most popular network architecture in the generation of medical images. Recently, residual network and transformer architectures have been utilized for medical image segmentation [54, 55]. Generative adversarial network framework [56] has the potential to improve performance to generate partial volume-corrected maps. Further studies applying these techniques for generating partial volume-corrected maps are required.

Another limitation of this study is that the features learned by the deepPVC model are too complicated for humans to understand. Therefore, the discussion on model learning in this study is speculative. However, the success of predicting PV-corrected maps observed in this study suggests that the deepPVC model learned some useful features for the correction of partial volume effects from MR and PET images. Further studies are required that interpret the model using techniques such as the attention mechanism [57, 58].

## Conclusions

We successfully predicted the PV-corrected maps using the deepPVC model trained with both MR and PiB PET images. The study results suggest that the deepPVC model learns useful features from the MR and PiB PET images, allowing the prediction of PV-corrected maps. However, the quantitative accuracy of PV-corrected maps predicted with deepPVC is imperfect compared to that of RBV PVC. Further improvement is required to ensure the accurate quantification of PV-corrected maps using deepPVC.

## Supplementary Information

**Additional file 1**: **Table S1**. List of FreeSurfer parcellation regions merged into each VOI in the present study. **Fig. S1** MR image and VOI map for a representative case. **Table S2** List for PET and MR scanners which acquired for subjects in training/validation and test dataset. **Fig. S2** Zoomed MR images and SUV maps around left frontal cortex for the representative cases of PiB-negative (top) and PiB-positive (bottom) shown in Figures 3 and 4 (bottom), respectively. The images on left to right indicate MR image, uncorrected PET image, SUV map PV-corrected by RBV, SUV map predicted by deepPVCMRI+PET, and SUV map predicted by deepPVCPET. Color ranges are same as Figure 3 and 4. **Fig. S3** Scatter plot (left) and Bland–Altman plot (right) between the real and predicted PV-corrected SUV on each VOI for the test data. Each dot indicates the regional SUV for one subject. The dashed line indicates perfect correspondence between the real and predicted SUVs. The red line indicates a regression line. **Fig. S4**. Trends of %differences in PV-corrected SUV on each region to the shifts and rotations for RBV PVC and deepPVC. Asterisks indicate significant differences between RBV PVC and deepPVC (paired *t* test; *p* < 0.05 (*); *p* < 0.001 (**)).

## Data Availability

The data used in this study are available from the ADNI database (http://adni.loni.usc.edu/) upon registration and compliance with the data usage agreement.

## Abbreviations

AD: Alzheimer’s disease
ADNI: Alzheimer’s Disease Neuroimaging Initiative
CNN: Convolutional neural network
FWHM: Full width half maximum
GTM: Geometric transfer matrix
ICC: Intraclass correlation
MCI: Mild cognitive impairment
MR-PVC: MR-guided partial volume correction
PET: Positron emission tomography
PoR: PVC-optimized registration
PSF: Point spread function
PVC: Partial volume correction
RBV: Region-based voxel-wise
ROI: Region of interest
SSIM: Structural similarity index
SUV: Standardized uptake value
SUVR: Standardized uptake value ratio
VOIs: Volumes of interest
