# Supplementary material for: DeepPVC: prediction of a partial volume-corrected map for brain positron emission tomography studies via a deep convolutional neural network
Source: EJNMMI Phys. 2022 Jul 30;9:50. doi: 10.1186/s40658-022-00478-8 (PMC9339068; doi:10.1186/s40658-022-00478-8)
Supplement: Supplementary file 1 — Additional file 1: Table S1. List of FreeSurfer parcellation regions merged into each VOI in the present study. Fig. S1 MR image and VOI map for a representative case. Table S2 List for PET and MR scanners which acquired for subjects in training/validation and test dataset. Fig. S2 Zoomed MR images and SUV maps around left frontal cortex for the representative cases of PiB-negative (top) and PiB-positive (bottom) shown in Figures 3 and 4 (bottom), respectively. The images on left to right indicate MR image, uncorrected PET image, SUV map PV-corrected by RBV, SUV map predicted by deepPVCMRI+PET, and SUV map predicted by deepPVCPET. Color ranges are same as Figure 3 and 4. Fig. S3 Scatter plot (left) and Bland–Altman plot (right) between the real and predicted PV-corrected SUV on each VOI for the test data. Each dot indicates the regional SUV for one subject. The dashed line indicates perfect correspondence between the real and predicted SUVs. The red line indicates a regression line. Fig. S4. Trends of %differences in PV-corrected SUV on each region to the shifts and rotations for RBV PVC and deepPVC. Asterisks indicate significant differences between RBV PVC and deepPVC (paired t test; p < 0.05 (*); p < 0.001 (**)). [file 40658_2022_478_MOESM1_ESM.pdf]

Supplementary Materials

Table S1. **List of FreeSurfer parcellation regions merged into each VOI in the present study.**

VOI	FreeSurfer parcellation
Frontal cortex	ctx-lh(rh)-caudalmiddlefrontal
	ctx-lh(rh)-lateralorbitofrontal
	ctx-lh(rh)-medialorbitofrontal
	ctx-lh(rh)-parsopercularis
	ctx-lh(rh)-parsorbitalis
	ctx-lh(rh)-parstriangularis
	ctx-lh(rh)-rostralmiddlefrontal
	ctx-lh(rh)-superiorfrontal
	ctx-lh(rh)-frontalpole
Parietal cortex	ctx-lh(rh)-inferiorparietal
	ctx-lh(rh)-superiorparietal
	ctx-lh(rh)-supramarginal
Precuneus	ctx-lh(rh)-precuneus
Occipital cortex	ctx-lh(rh)-cuneus
	ctx-lh(rh)-lateraloccipital
	ctx-lh(rh)-lingual
	ctx-lh(rh)-pericalcarine
Lateral temporal cortex	ctx-lh(rh)-bankssts
	ctx-lh(rh)-inferiortemporal
	ctx-lh(rh)-middletemporal
	ctx-lh(rh)-superiortemporal
	ctx-lh(rh)-temporalpole
	ctx-lh(rh)-transversetemporal
Medial temporal cortex	ctx-lh(rh)-enthorhinal
	ctx-lh(rh)-fusiform
	ctx-lh(rh)-parahippocampal

Anterior cingulate cortex	ctx-lh(rh)-caudalanteriorcingulate
	ctx-lh(rh)-rostralanteriorcingulate
Posterior cingulate cortex	ctx-lh(rh)-isthmuscingulate
	ctx-lh(rh)-posteriorcingulate
Sensory motor cortex	ctx-lh(rh)-paracentral
	ctx-lh(rh)-postcentral
	ctx-lh(rh)-precentral
Insula	ctx-lh(rh)-insula
Caudate	Caudate
Putamen	Putamen
Thalamus	Thalamus-Proper
	Thalamus
	VentralDC
Accumbens area	Accumbens area
Pallidum	Pallidum
Brainstem	Brainstem
Hippocampus	Hippocampus
Amygdala	Amygdala
Cerebellar gray matter	Cerebellum–Cortex
Subcortical white matter	Cerebral white matter
	WM hypointensities
	CC_Posterior
	CC_Mid_Posterior
	CC_Central
	CC_Mid_Anterior
	CC_Anterior
Cerebellar white matter	Cerebellum–white matter
Cerebrospinal fluid	Lateral ventricle
	Inf–Lat–Vent
	3rd ventricle
	4th ventricle
	CSF

	vessel
	choroid plexus
	5th ventricle

The 113 regions parcellated using FreeSurfer (aparc + aseg) were merged into 44 regions (22 regions on each hemisphere) for region-based PVC and VOI analysis in this study. MR image and VOI map for a representative case are shown in Figure S1.

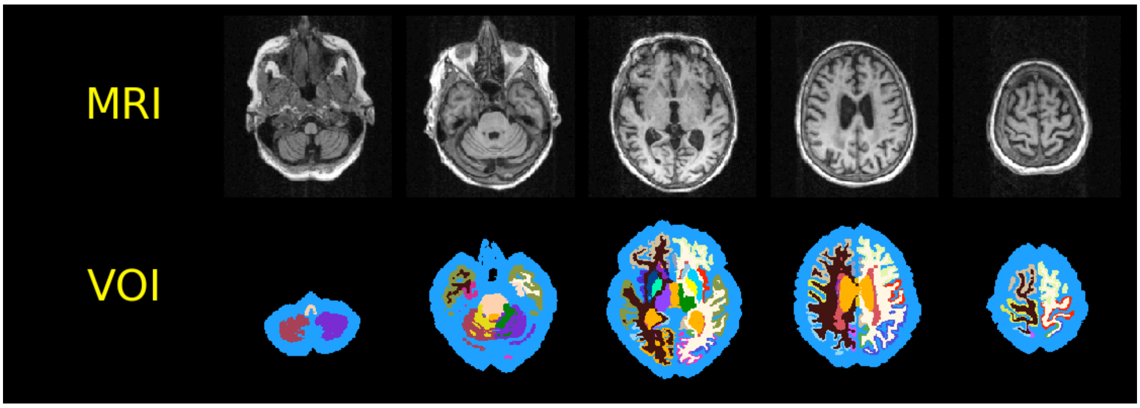

Figure S1 MR image and VOI map for a representative case.

Table S2 List for PET and MR scanners which acquired for subjects in training/validation and test dataset.

	PET scanner [manufacturer]	MR scanner [manufacturer]	No. of subjects
Training/validation	Discovery RX [GE]	SIGNA HDx [GE]	10
		SIGNA HDxt [GE]	4
	ECAT HR+ [Siemens]	Genesis SIGNA [GE]	2
		Intera [Philips]	8
		SIGNA EXCITE [GE]	33
		SIGNA HDx [GE]	16
		Sonata [Siemens]	35
	HRRT [Siemens]	Avanto [Siemens]	44
		Intera [Philips]	4
Test [PiB]	Advance [GE]	Avanto [Siemens]	1
		Intera [Philips]	3
		SIGNA HDx	32
Test [FDG]	HRRT [Siemens]	SIGNA EXCITE [GE]	6
		SIGNA HDx	10

Figure S2 Zoomed MR images and SUV maps around left frontal cortex for the representative cases of PiB-negative (top) and PiB-positive (bottom) shown in Figure 3 and 4 (bottom), respectively. The images on left to right indicate MR image, uncorrected PET image, SUV map PV-corrected by RBV, SUV map predicted by deepPVC$_{MRI+PET}$, and SUV map predicted by deepPVC$_{PET}$. Color ranges are same as Figure 3 and 4.

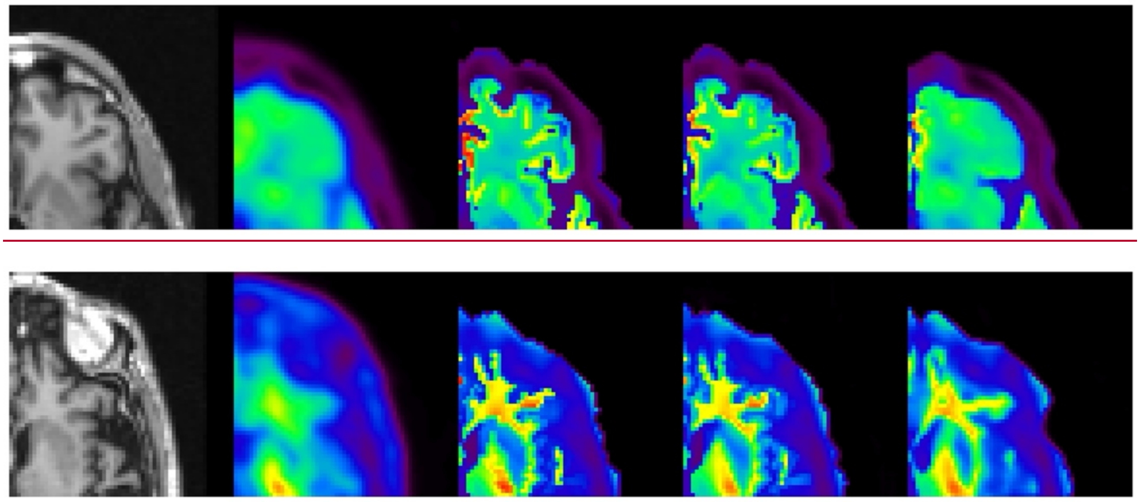

Figure S3 Scatter plot (left) and Bland-Altman plot (right) between the real and predicted PV-corrected SUV on each VOI for the test data. Each dot indicates the regional SUV for one subject. The dashed line indicates perfect correspondence between the real and predicted SUVs. The red line indicates a regression line.

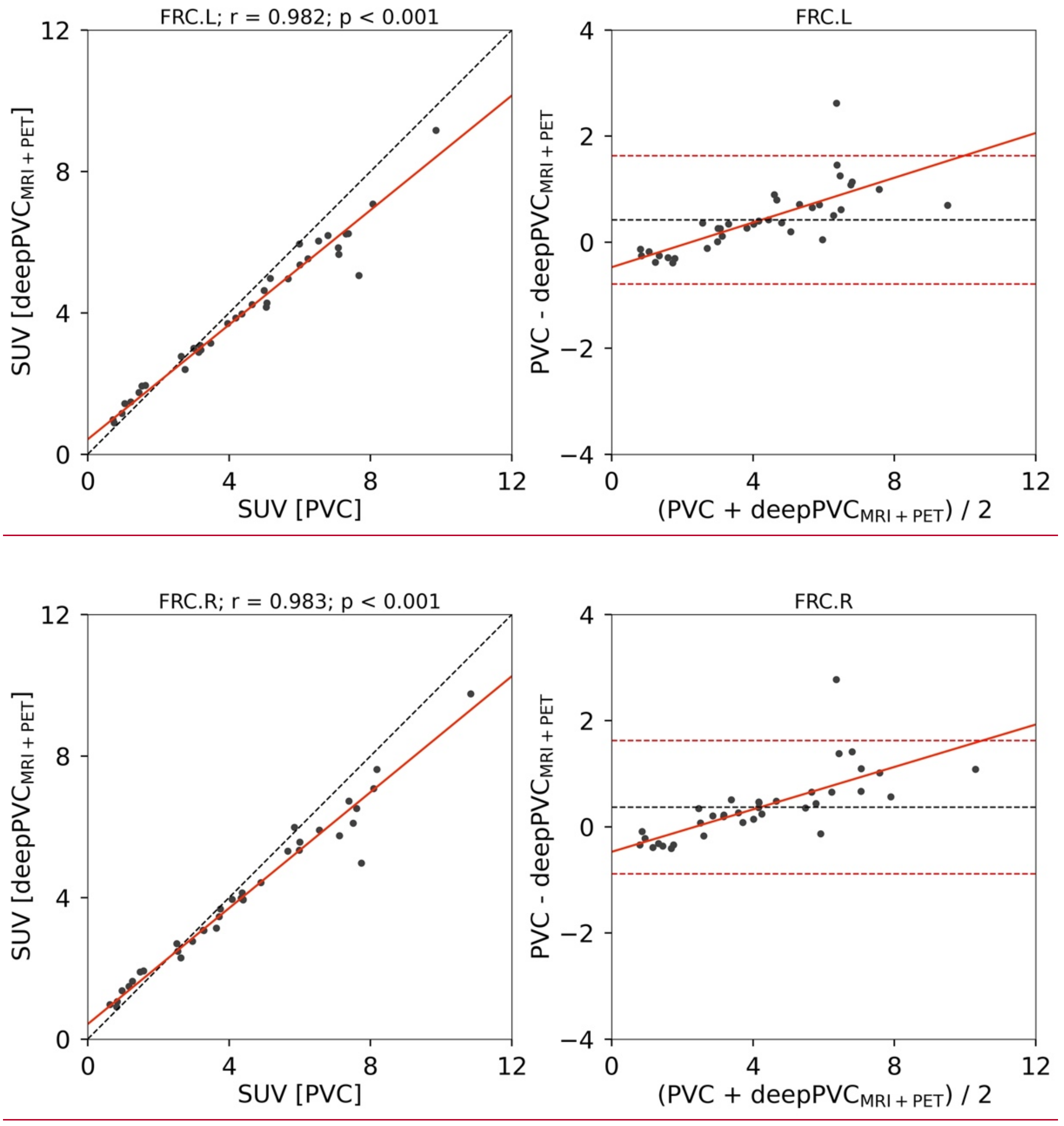

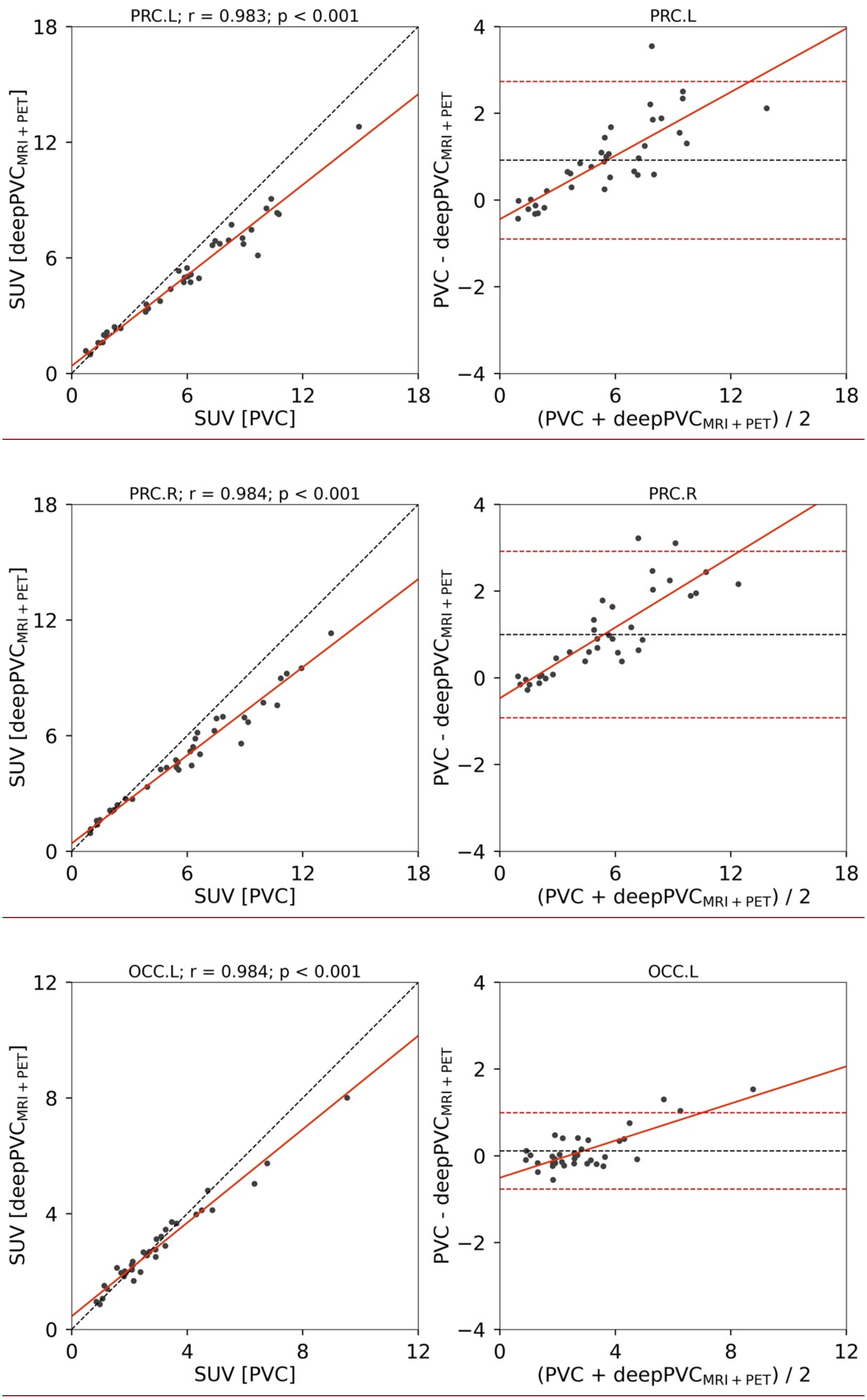

PRC.L; r = 0.983; p < 0.001
SUV [deepPVC_MRI + PET]
SUV [PVC]
PRC.L
PVC - deepPVC_MRI + PET
(PVC + deepPVC_MRI + PET) / 2
PRC.R; r = 0.984; p < 0.001
SUV [deepPVC_MRI + PET]
SUV [PVC]
PRC.R
PVC - deepPVC_MRI + PET
(PVC + deepPVC_MRI + PET) / 2
OCC.L; r = 0.984; p < 0.001
SUV [deepPVC_MRI + PET]
SUV [PVC]
OCC.L
PVC - deepPVC_MRI + PET
(PVC + deepPVC_MRI + PET) / 2

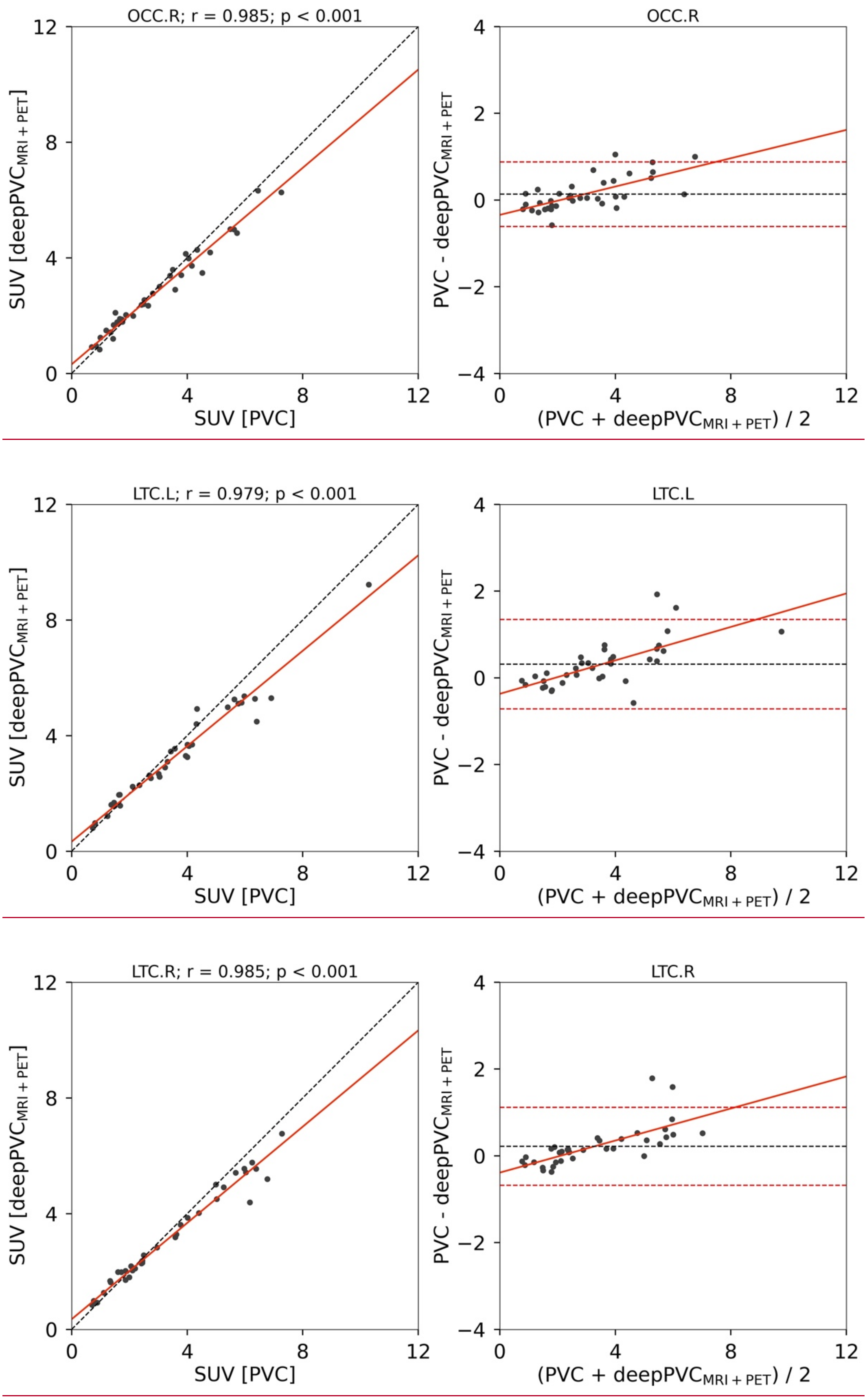
OCC.R; r = 0.985; p < 0.001
SUV [deepPVC$_{MRI + PET}$]
SUV [PVC]
OCC.R
PVC - deepPVC$_{MRI + PET}$
(PVC + deepPVC$_{MRI + PET}$) / 2
LTC.L; r = 0.979; p < 0.001
SUV [deepPVC$_{MRI + PET}$]
SUV [PVC]
LTC.L
PVC - deepPVC$_{MRI + PET}$
(PVC + deepPVC$_{MRI + PET}$) / 2
LTC.R; r = 0.985; p < 0.001
SUV [deepPVC$_{MRI + PET}$]
SUV [PVC]
LTC.R
PVC - deepPVC$_{MRI + PET}$
(PVC + deepPVC$_{MRI + PET}$) / 2

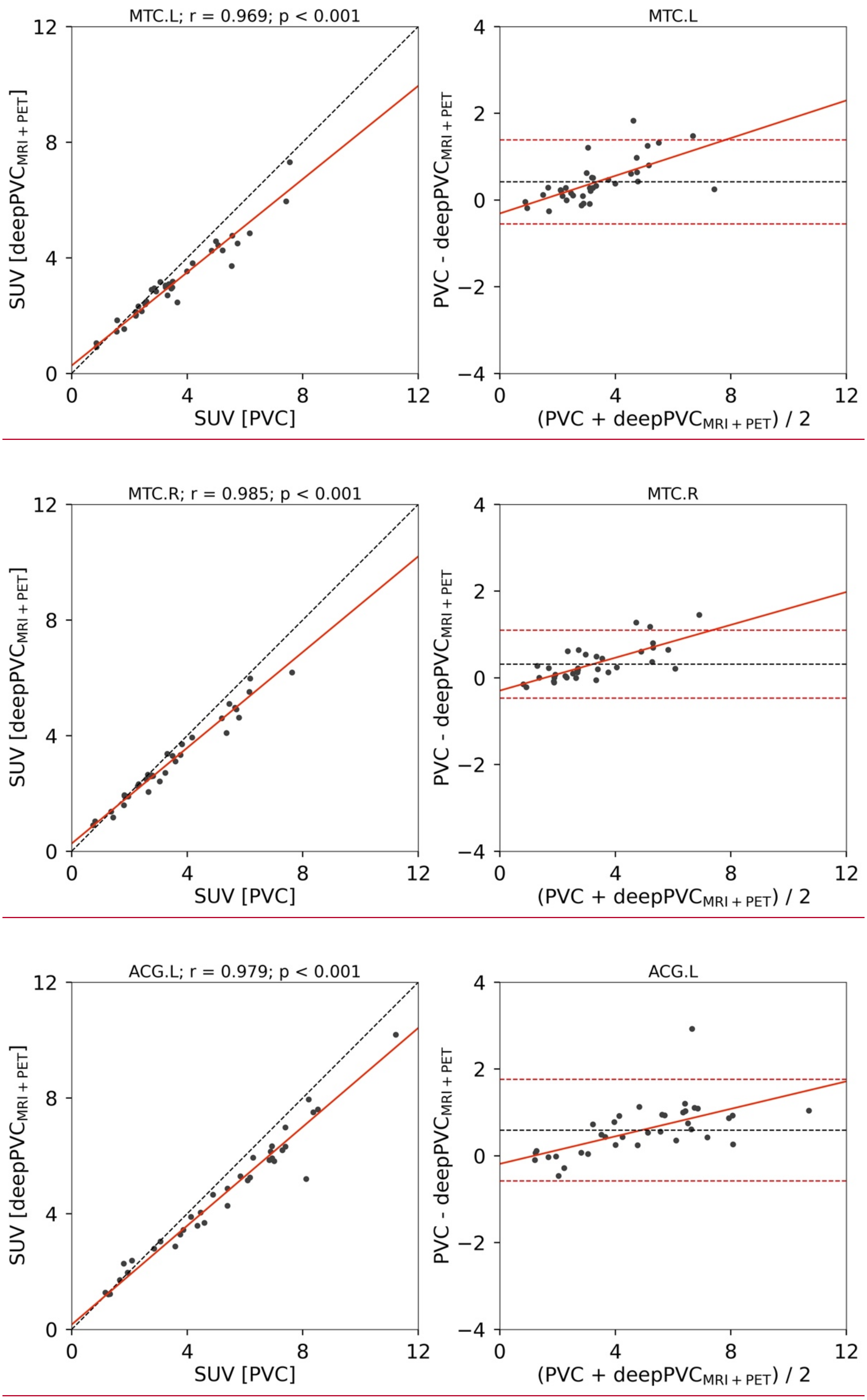
MTC.L; r = 0.969; p < 0.001
SUV [deepPVC$_{MRI+PET}$]
SUV [PVC]
MTC.L
PVC - deepPVC$_{MRI+PET}$
(PVC + deepPVC$_{MRI+PET}$) / 2
MTC.R; r = 0.985; p < 0.001
SUV [deepPVC$_{MRI+PET}$]
SUV [PVC]
MTC.R
PVC - deepPVC$_{MRI+PET}$
(PVC + deepPVC$_{MRI+PET}$) / 2
ACG.L; r = 0.979; p < 0.001
SUV [deepPVC$_{MRI+PET}$]
SUV [PVC]
ACG.L
PVC - deepPVC$_{MRI+PET}$
(PVC + deepPVC$_{MRI+PET}$) / 2

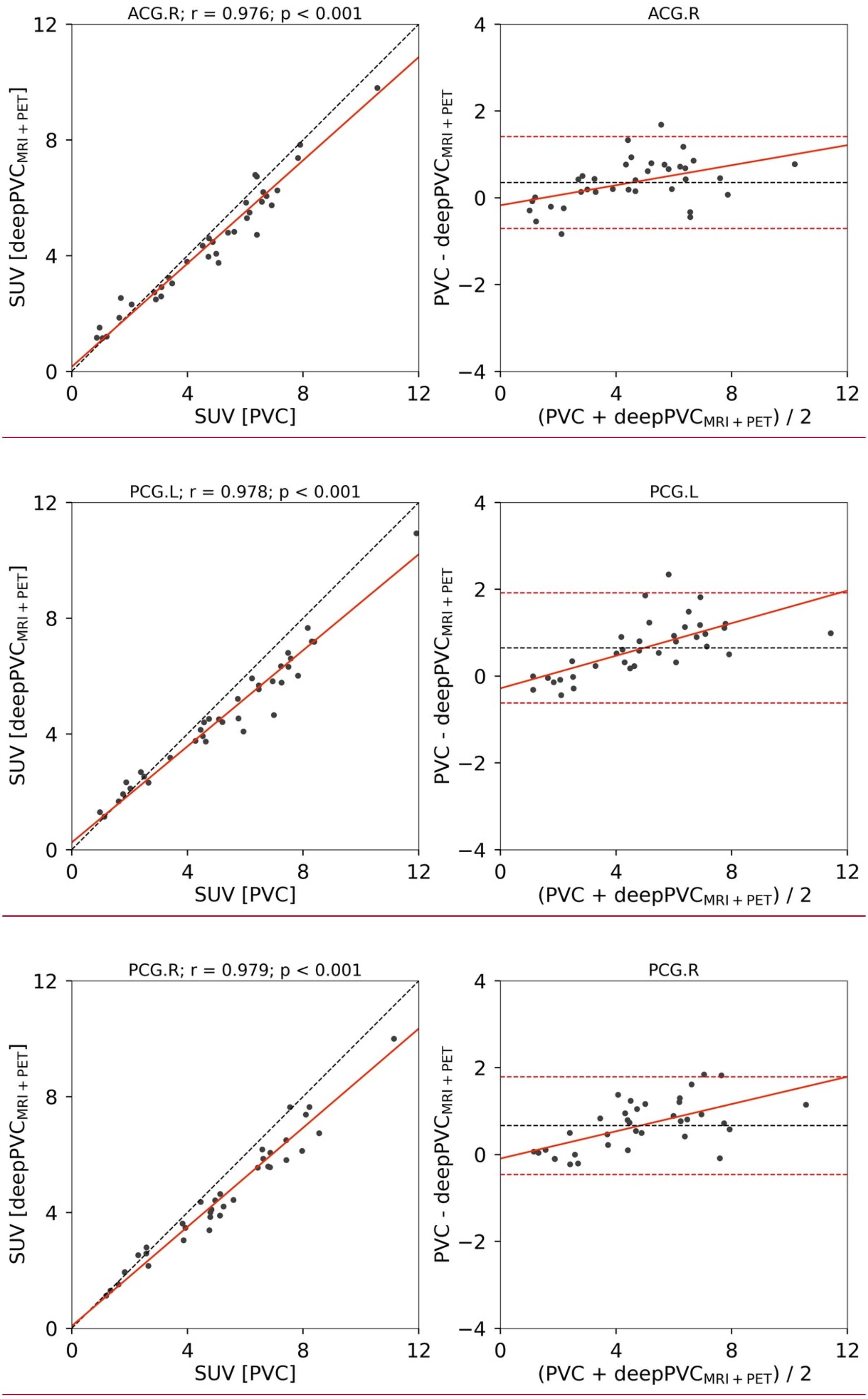

ACG.R; r = 0.976; p < 0.001
SUV [deepPVC$_{MRI+PET}$]
SUV [PVC]
ACG.R
PVC - deepPVC$_{MRI+PET}$
(PVC + deepPVC$_{MRI+PET}$) / 2
PCG.L; r = 0.978; p < 0.001
SUV [deepPVC$_{MRI+PET}$]
SUV [PVC]
PCG.L
PVC - deepPVC$_{MRI+PET}$
(PVC + deepPVC$_{MRI+PET}$) / 2
PCG.R; r = 0.979; p < 0.001
SUV [deepPVC$_{MRI+PET}$]
SUV [PVC]
PCG.R
PVC - deepPVC$_{MRI+PET}$
(PVC + deepPVC$_{MRI+PET}$) / 2

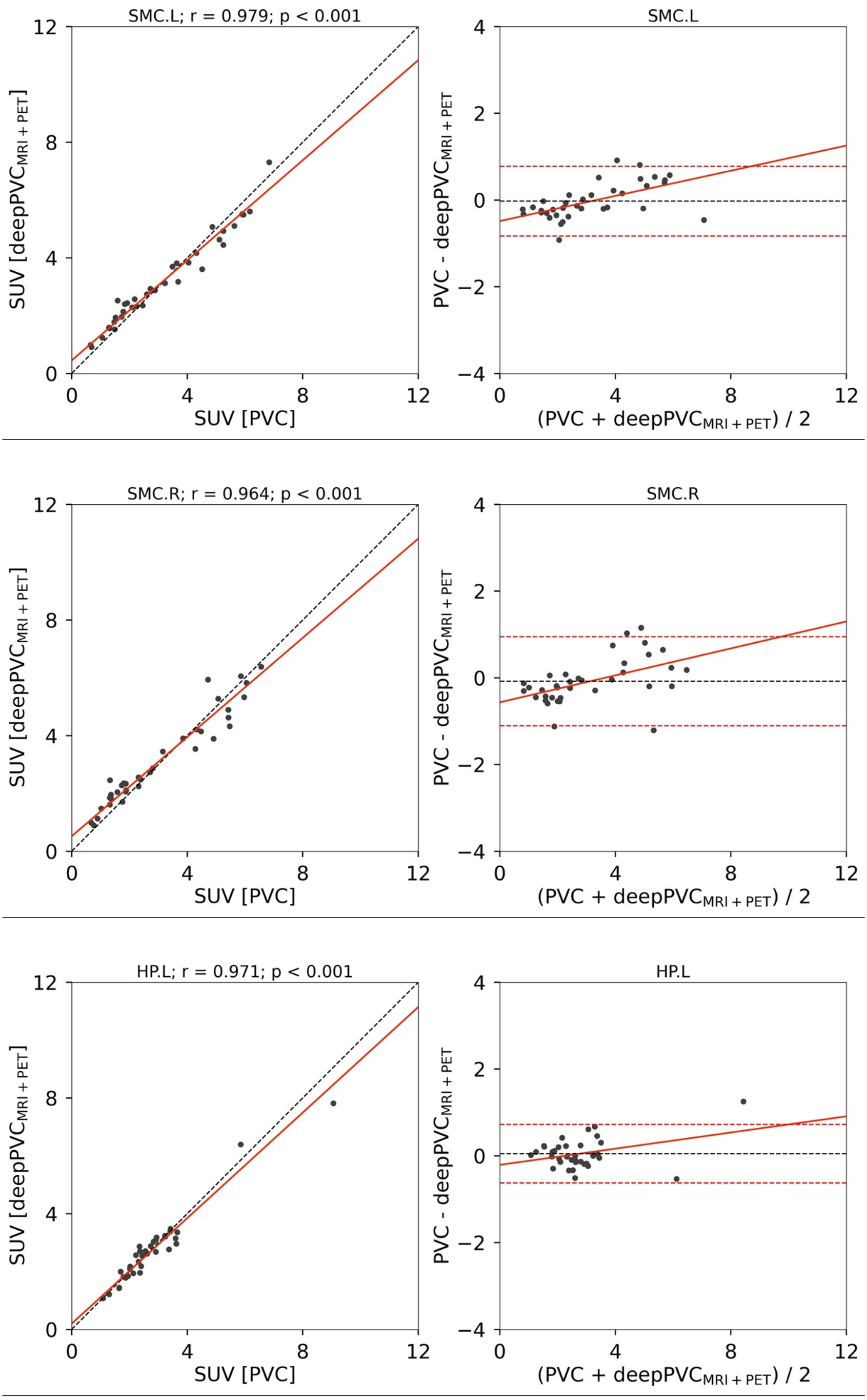

SMC.L; r = 0.979; p < 0.001
SUV [deepPVC$_{MRI+PET}$]
SUV [PVC]
SMC.L
PVC - deepPVC$_{MRI+PET}$
(PVC + deepPVC$_{MRI+PET}$) / 2
SMC.R; r = 0.964; p < 0.001
SUV [deepPVC$_{MRI+PET}$]
SUV [PVC]
SMC.R
PVC - deepPVC$_{MRI+PET}$
(PVC + deepPVC$_{MRI+PET}$) / 2
HP.L; r = 0.971; p < 0.001
SUV [deepPVC$_{MRI+PET}$]
SUV [PVC]
HP.L
PVC - deepPVC$_{MRI+PET}$
(PVC + deepPVC$_{MRI+PET}$) / 2

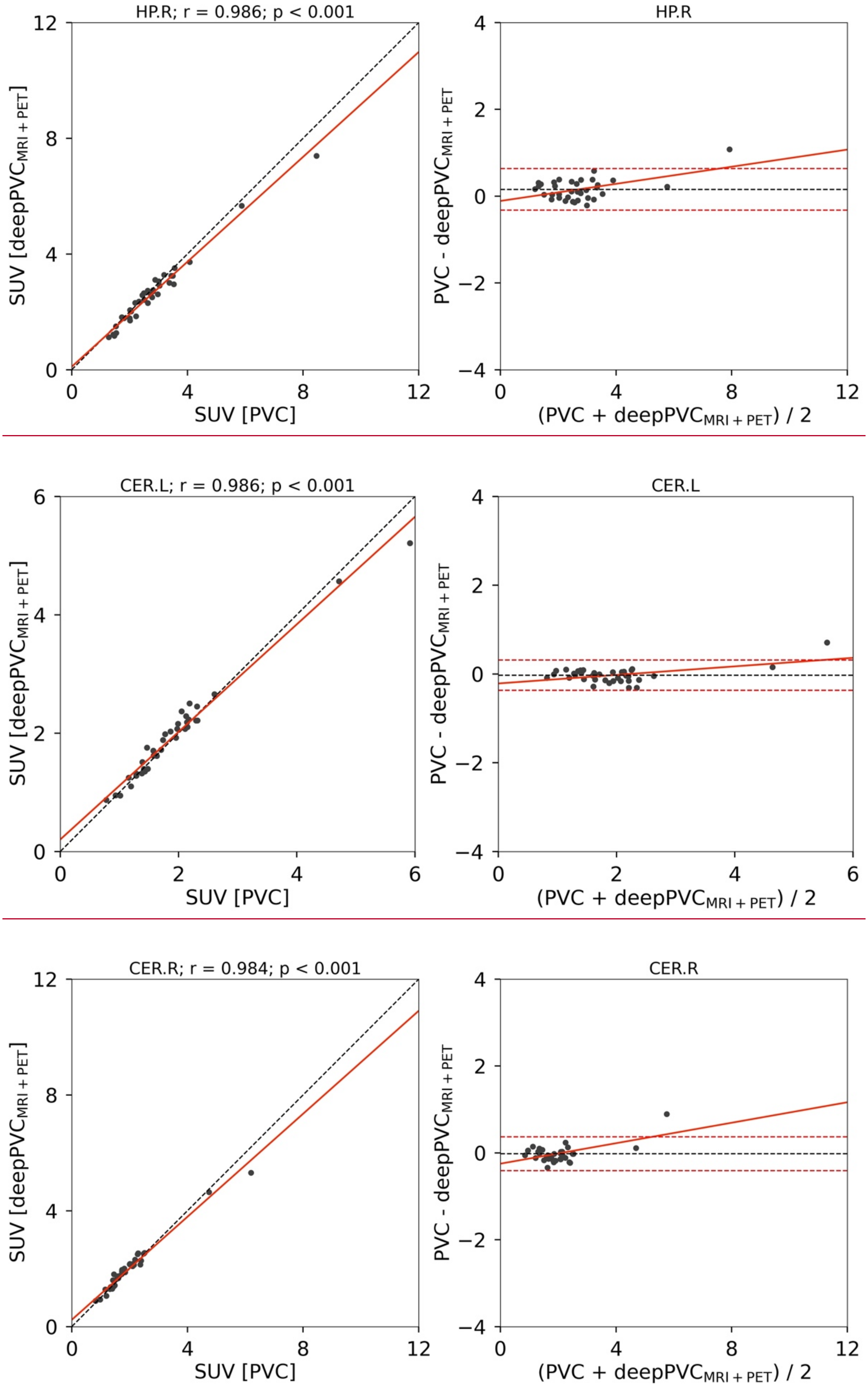
HP.R; r = 0.986; p < 0.001
SUV [deepPVC$_{MRI+PET}$]
SUV [PVC]
HP.R
PVC - deepPVC$_{MRI+PET}$
(PVC + deepPVC$_{MRI+PET}$) / 2
CER.L; r = 0.986; p < 0.001
SUV [deepPVC$_{MRI+PET}$]
SUV [PVC]
CER.L
PVC - deepPVC$_{MRI+PET}$
(PVC + deepPVC$_{MRI+PET}$) / 2
CER.R; r = 0.984; p < 0.001
SUV [deepPVC$_{MRI+PET}$]
SUV [PVC]
CER.R
PVC - deepPVC$_{MRI+PET}$
(PVC + deepPVC$_{MRI+PET}$) / 2

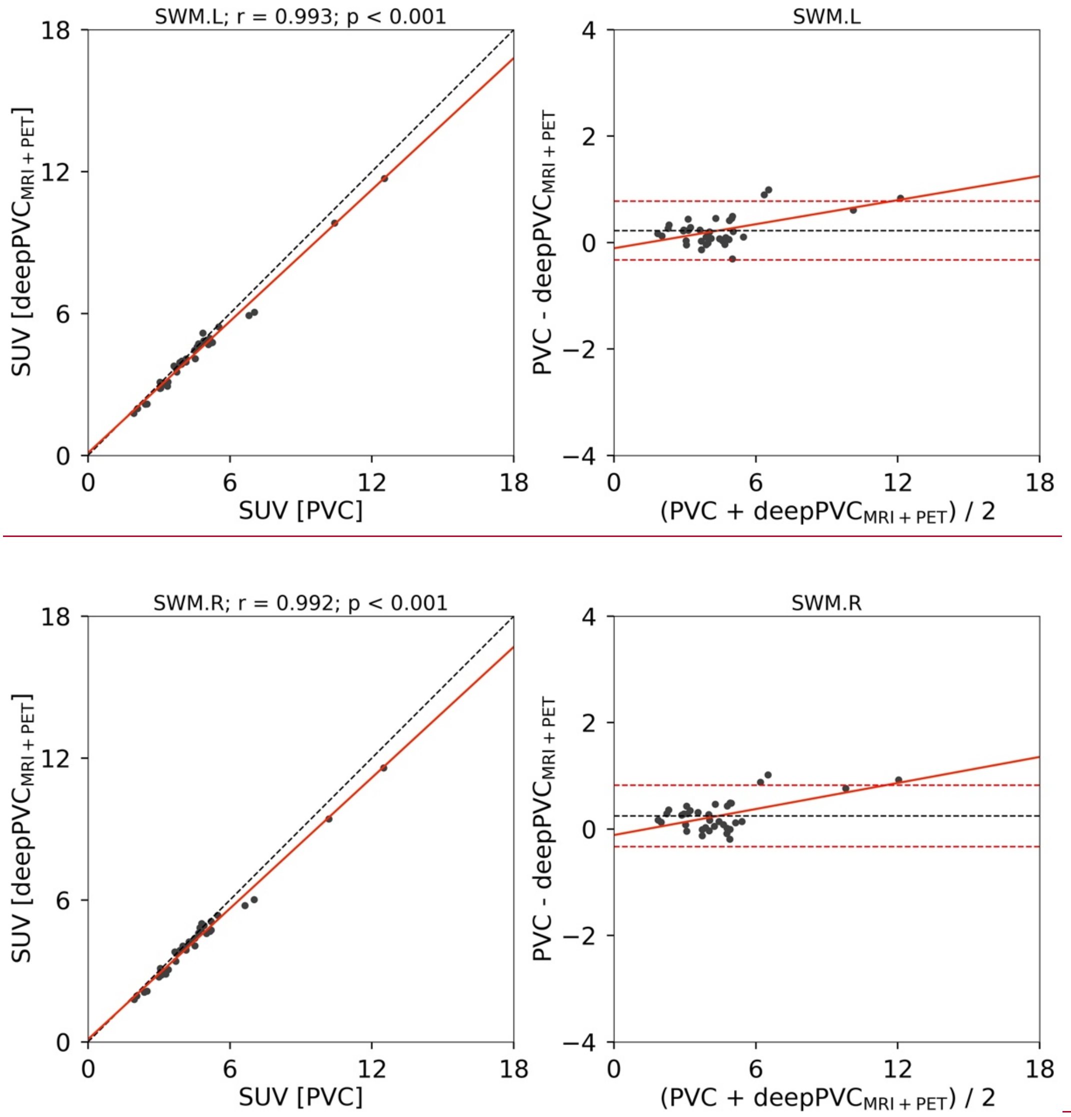
SWM.L; r = 0.993; p < 0.001
SUV [deepPVC$_{MRI+PET}$]
SUV [PVC]
SWM.L
PVC - deepPVC$_{MRI+PET}$
(PVC + deepPVC$_{MRI+PET}$) / 2
SWM.R; r = 0.992; p < 0.001
SUV [deepPVC$_{MRI+PET}$]
SUV [PVC]
SWM.R
PVC - deepPVC$_{MRI+PET}$
(PVC + deepPVC$_{MRI+PET}$) / 2

Figure ~~S2~~ S4 Trends of %differences in PV-corrected SUV on each region to the shifts and rotations for RBV PVC and deepPVC. Asterisks indicate significant differences between RBV PVC and deepPVC (paired *t*-test; $p < 0.05$ (*); p < 0.001 (**)).

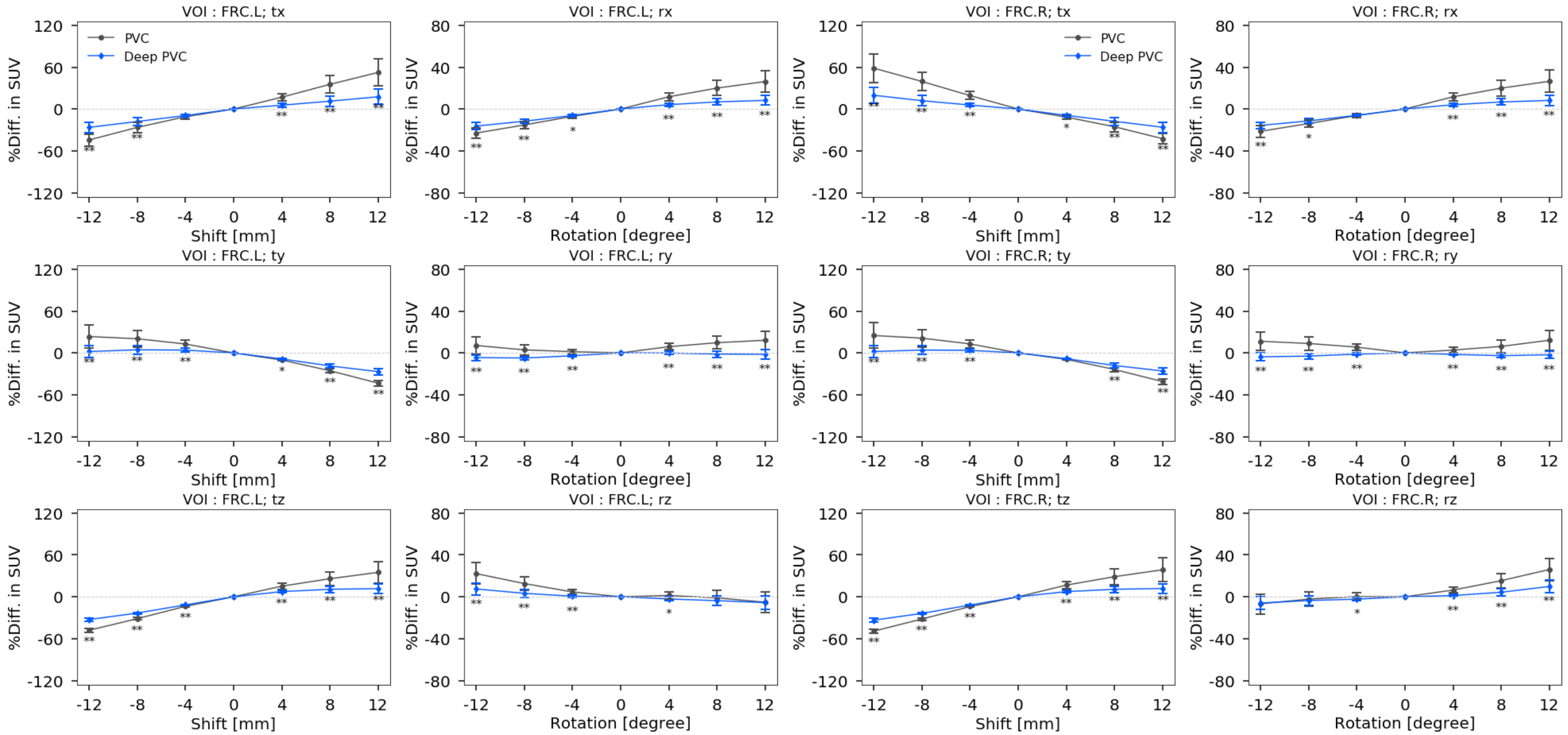

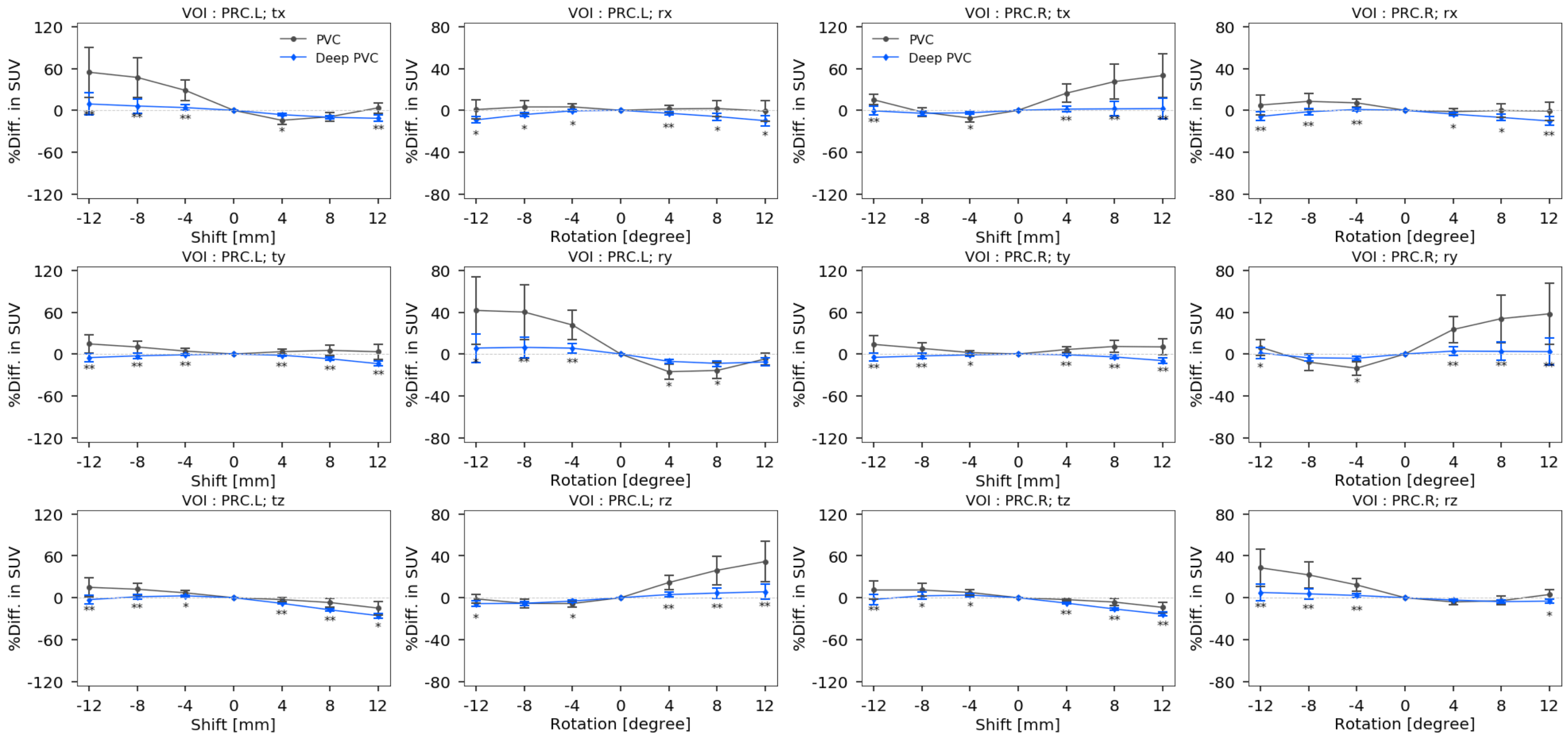

VOI : PRC.L; tx
VOI : PRC.L; rx
VOI : PRC.R; tx
VOI : PRC.R; rx
VOI : PRC.L; ty
VOI : PRC.L; ry
VOI : PRC.R; ty
VOI : PRC.R; ry
VOI : PRC.L; tz
VOI : PRC.L; rz
VOI : PRC.R; tz
VOI : PRC.R; rz
PVC
Deep PVC
%Diff. in SUV
Shift [mm]
Rotation [degree]

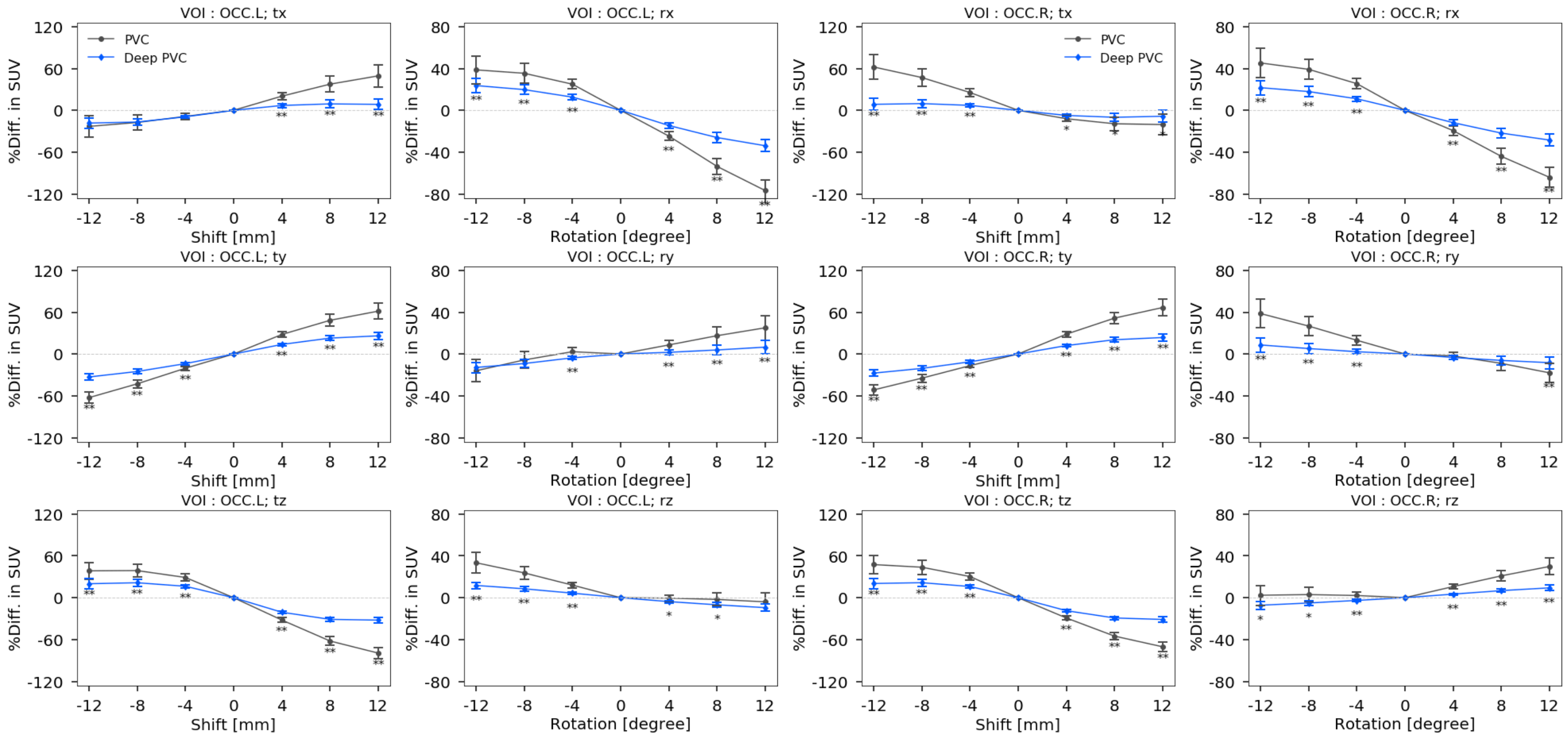
VOI : OCC.L; tx
VOI : OCC.L; rx
VOI : OCC.R; tx
VOI : OCC.R; rx
VOI : OCC.L; ty
VOI : OCC.L; ry
VOI : OCC.R; ty
VOI : OCC.R; ry
VOI : OCC.L; tz
VOI : OCC.L; rz
VOI : OCC.R; tz
VOI : OCC.R; rz
PVC
Deep PVC
%Diff. in SUV
Shift [mm]
Rotation [degree]

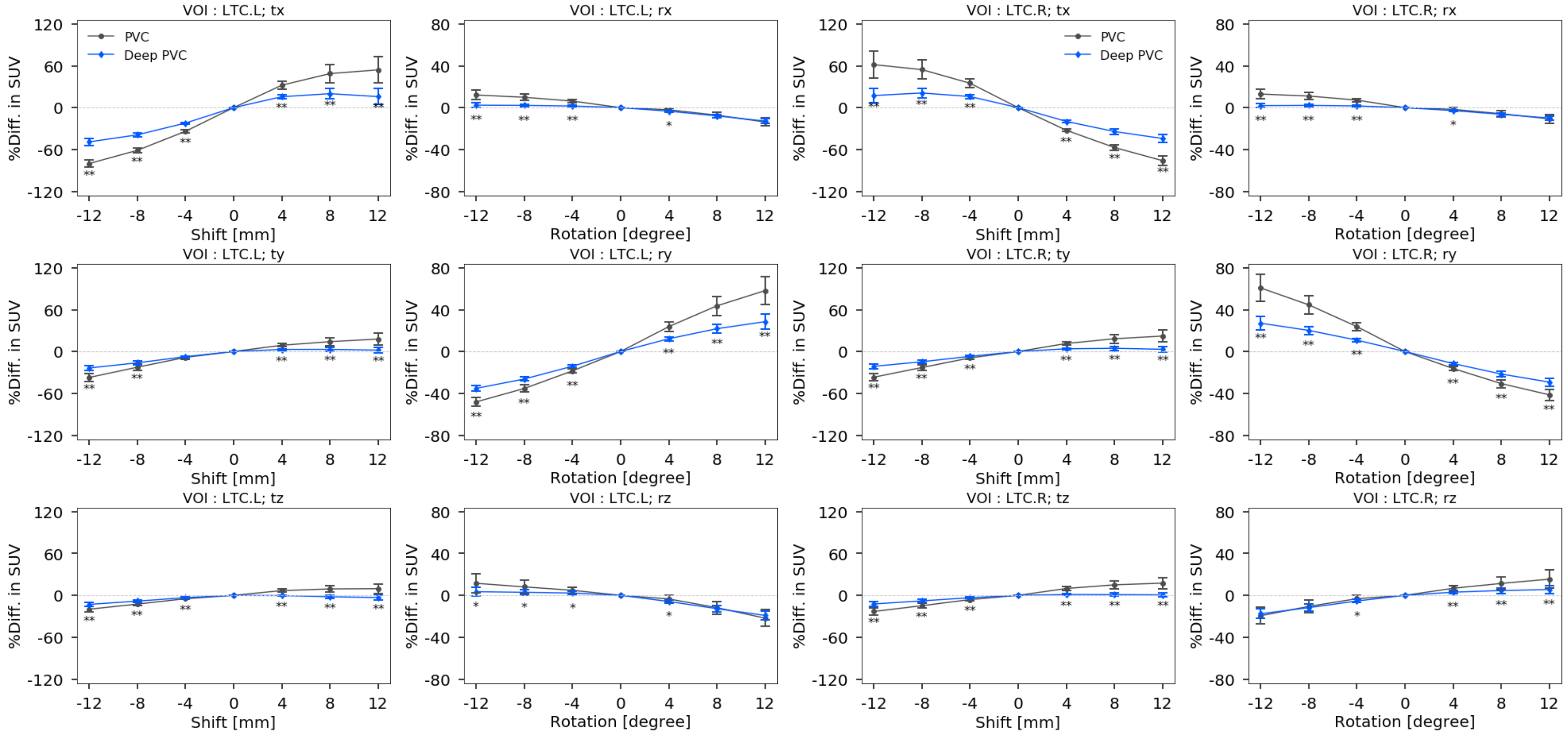
VOI : LTC.L; tx
VOI : LTC.L; rx
VOI : LTC.R; tx
VOI : LTC.R; rx
VOI : LTC.L; ty
VOI : LTC.L; ry
VOI : LTC.R; ty
VOI : LTC.R; ry
VOI : LTC.L; tz
VOI : LTC.L; rz
VOI : LTC.R; tz
VOI : LTC.R; rz
PVC
Deep PVC
%Diff. in SUV
Shift [mm]
Rotation [degree]

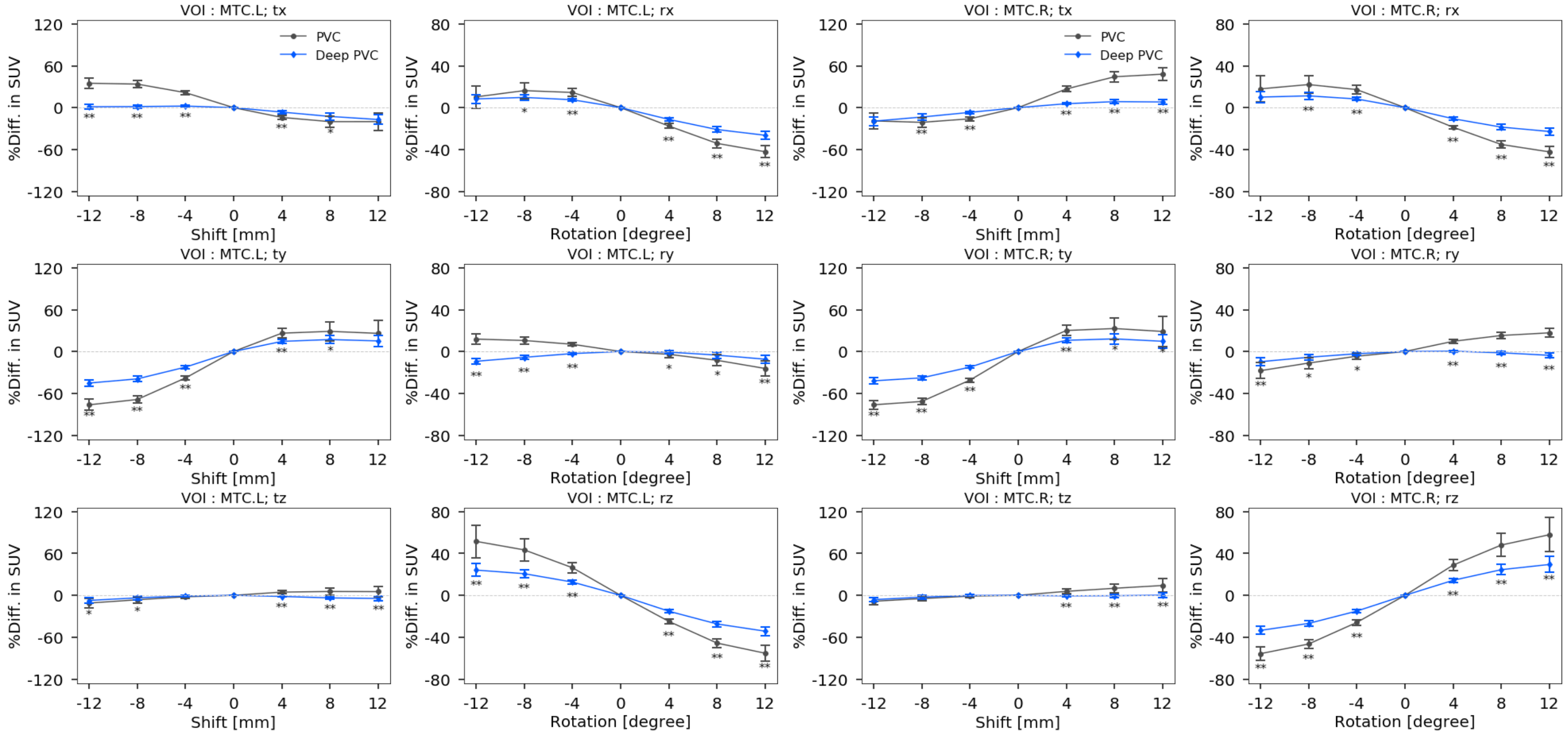
VOI : MTC.L; tx
VOI : MTC.L; rx
VOI : MTC.R; tx
VOI : MTC.R; rx
VOI : MTC.L; ty
VOI : MTC.L; ry
VOI : MTC.R; ty
VOI : MTC.R; ry
VOI : MTC.L; tz
VOI : MTC.L; rz
VOI : MTC.R; tz
VOI : MTC.R; rz
PVC
Deep PVC
%Diff. in SUV
Shift [mm]
Rotation [degree]

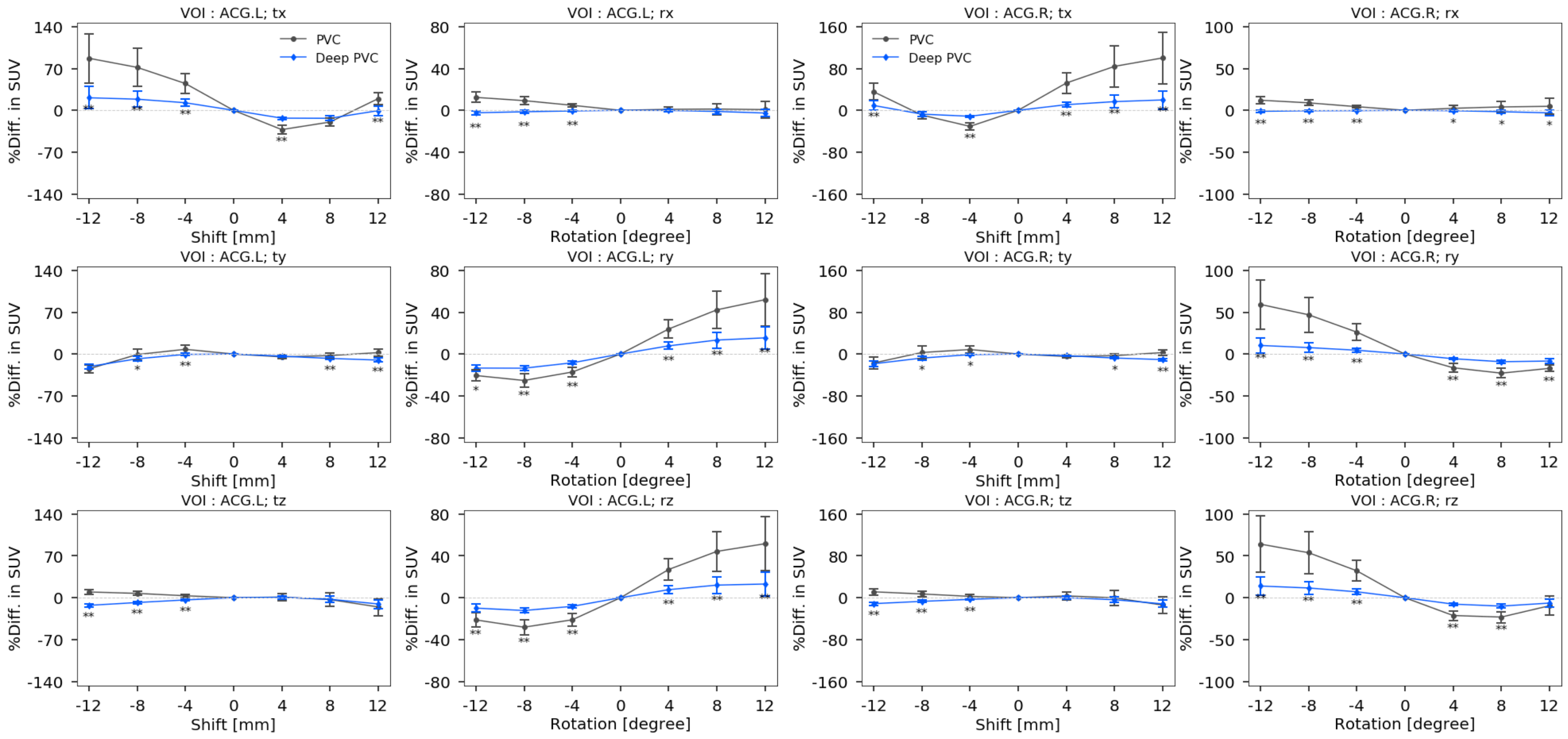
VOI : ACG.L; tx
VOI : ACG.L; rx
VOI : ACG.R; tx
VOI : ACG.R; rx
VOI : ACG.L; ty
VOI : ACG.L; ry
VOI : ACG.R; ty
VOI : ACG.R; ry
VOI : ACG.L; tz
VOI : ACG.L; rz
VOI : ACG.R; tz
VOI : ACG.R; rz
PVC
Deep PVC
%Diff. in SUV
Shift [mm]
Rotation [degree]

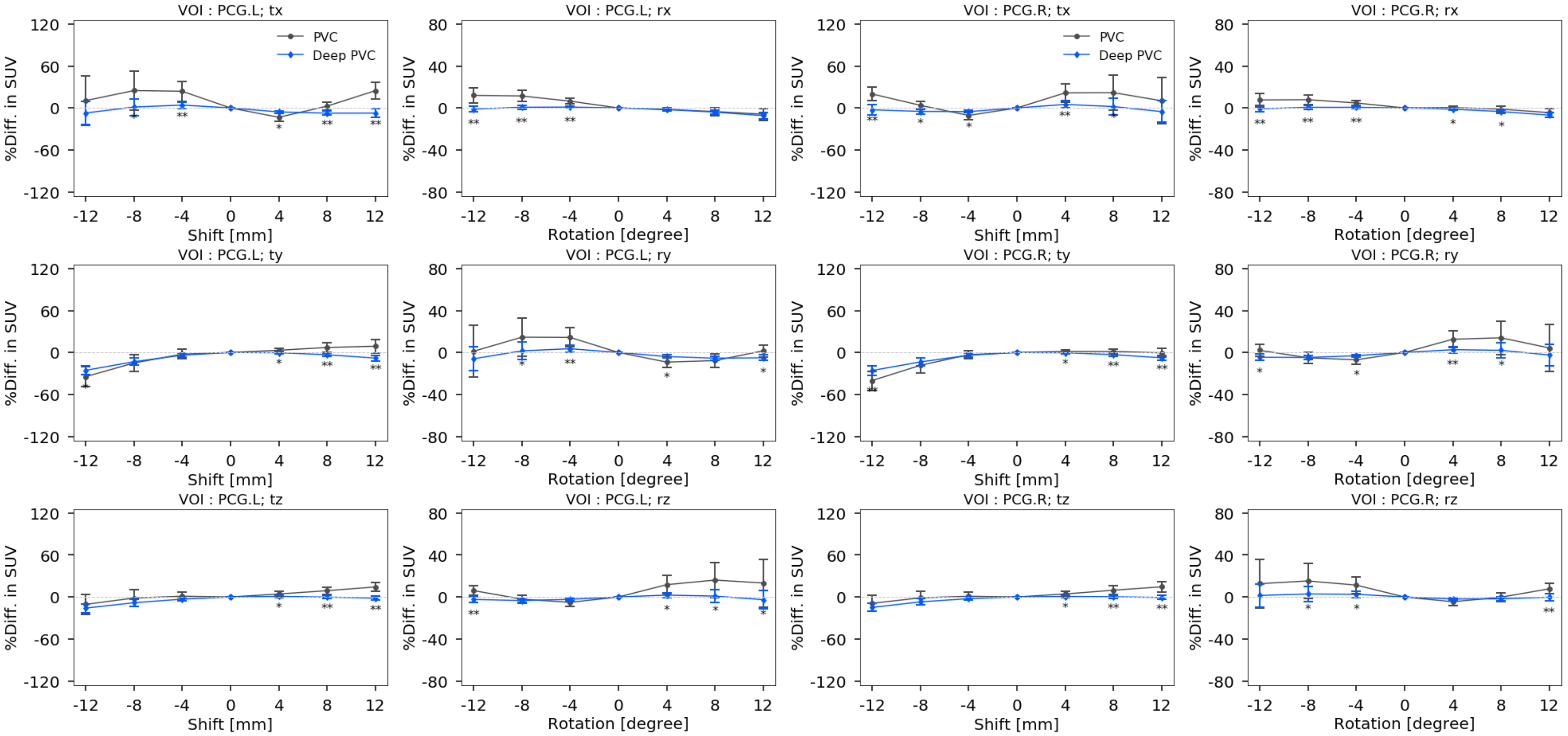
VOI : PCG.L; tx
VOI : PCG.L; rx
VOI : PCG.R; tx
VOI : PCG.R; rx
VOI : PCG.L; ty
VOI : PCG.L; ry
VOI : PCG.R; ty
VOI : PCG.R; ry
VOI : PCG.L; tz
VOI : PCG.L; rz
VOI : PCG.R; tz
VOI : PCG.R; rz
PVC
Deep PVC
%Diff. in SUV
Shift [mm]
Rotation [degree]
-12 -8 -4 0 4 8 12
120 60 0 -60 -120
80 40 0 -40 -80

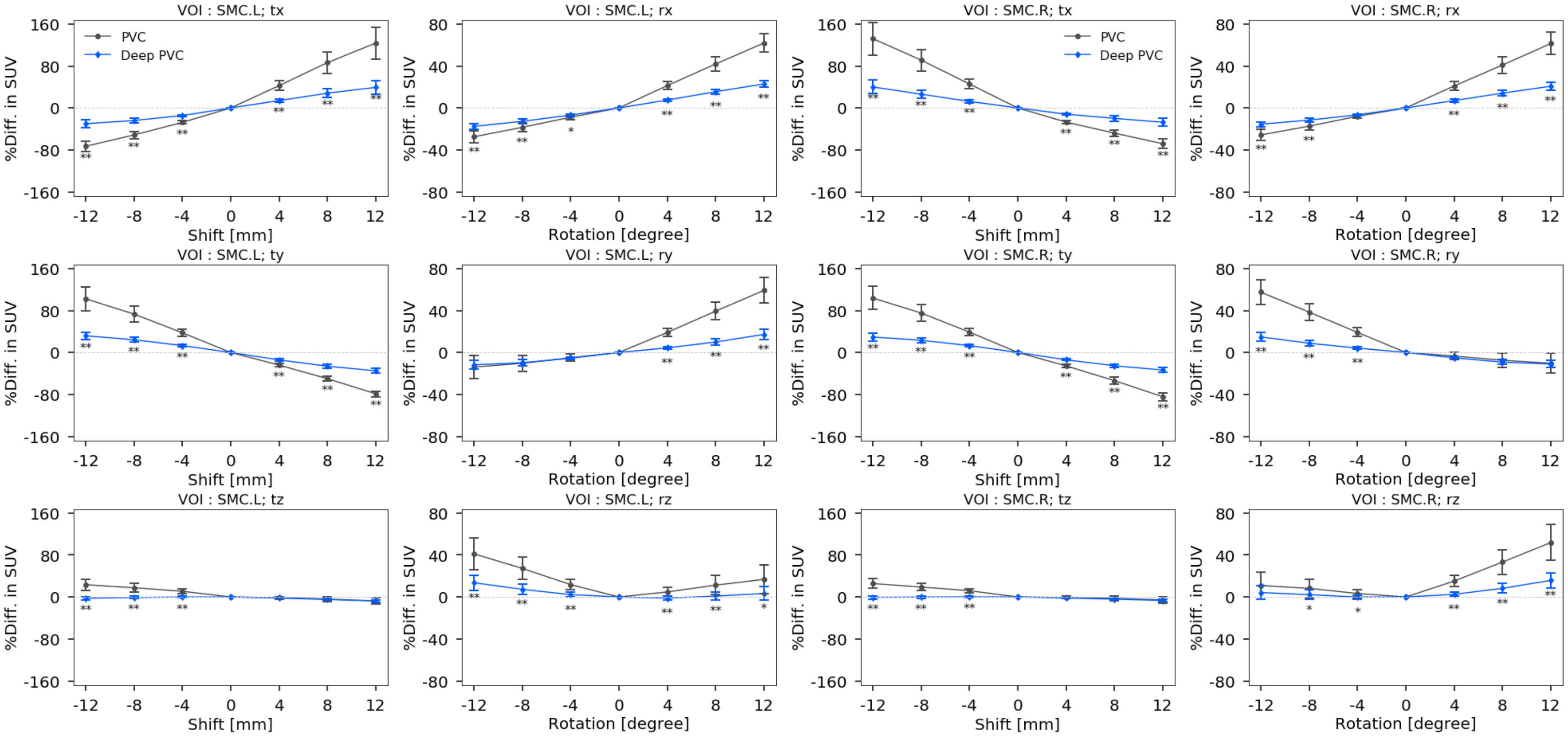
VOI : SMC.L; tx
VOI : SMC.L; rx
VOI : SMC.R; tx
VOI : SMC.R; rx
VOI : SMC.L; ty
VOI : SMC.L; ry
VOI : SMC.R; ty
VOI : SMC.R; ry
VOI : SMC.L; tz
VOI : SMC.L; rz
VOI : SMC.R; tz
VOI : SMC.R; rz
PVC
Deep PVC
%Diff. in SUV
Shift [mm]
Rotation [degree]

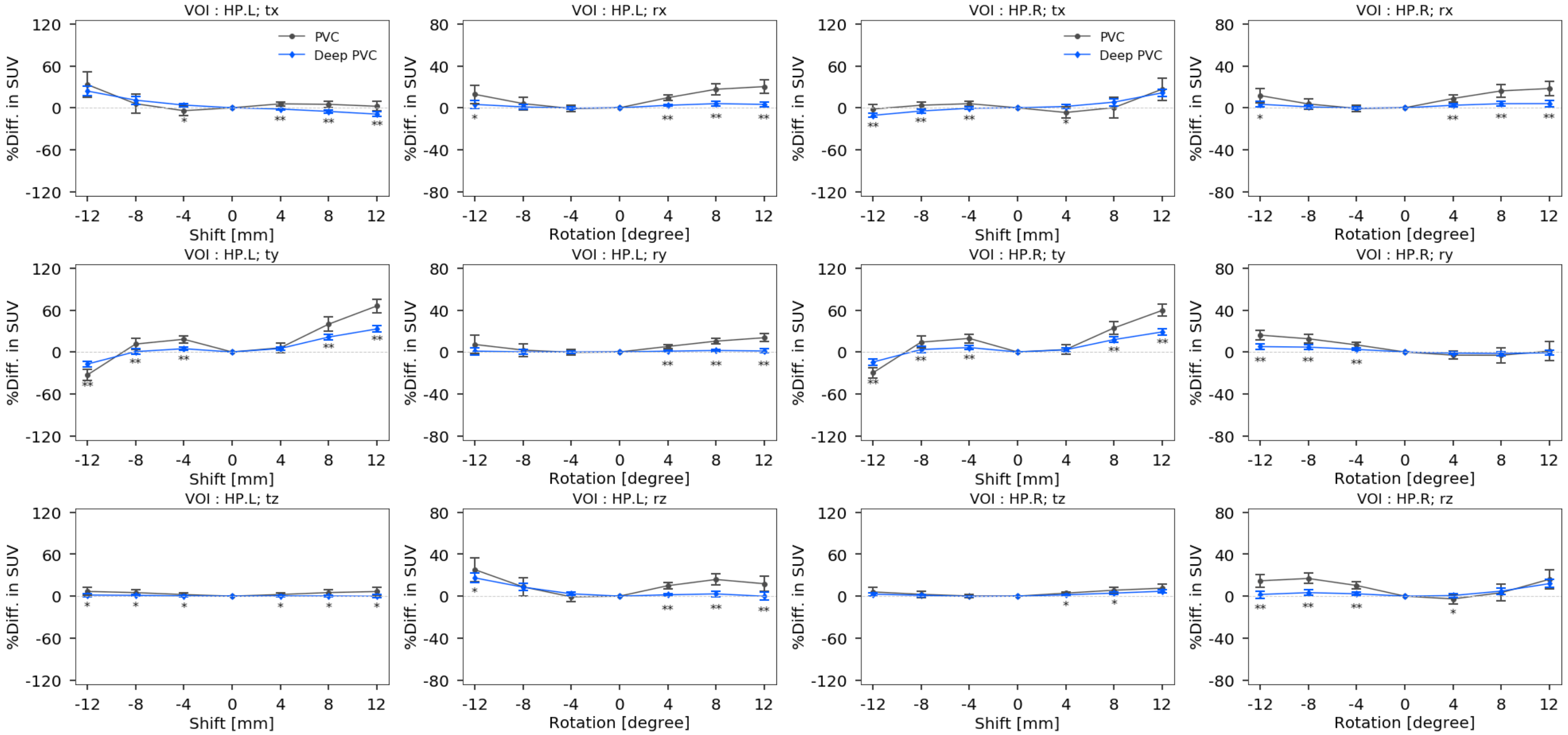

VOI : HP.L; tx
VOI : HP.L; rx
VOI : HP.R; tx
VOI : HP.R; rx
VOI : HP.L; ty
VOI : HP.L; ry
VOI : HP.R; ty
VOI : HP.R; ry
VOI : HP.L; tz
VOI : HP.L; rz
VOI : HP.R; tz
VOI : HP.R; rz
PVC
Deep PVC
%Diff. in SUV
Shift [mm]
Rotation [degree]

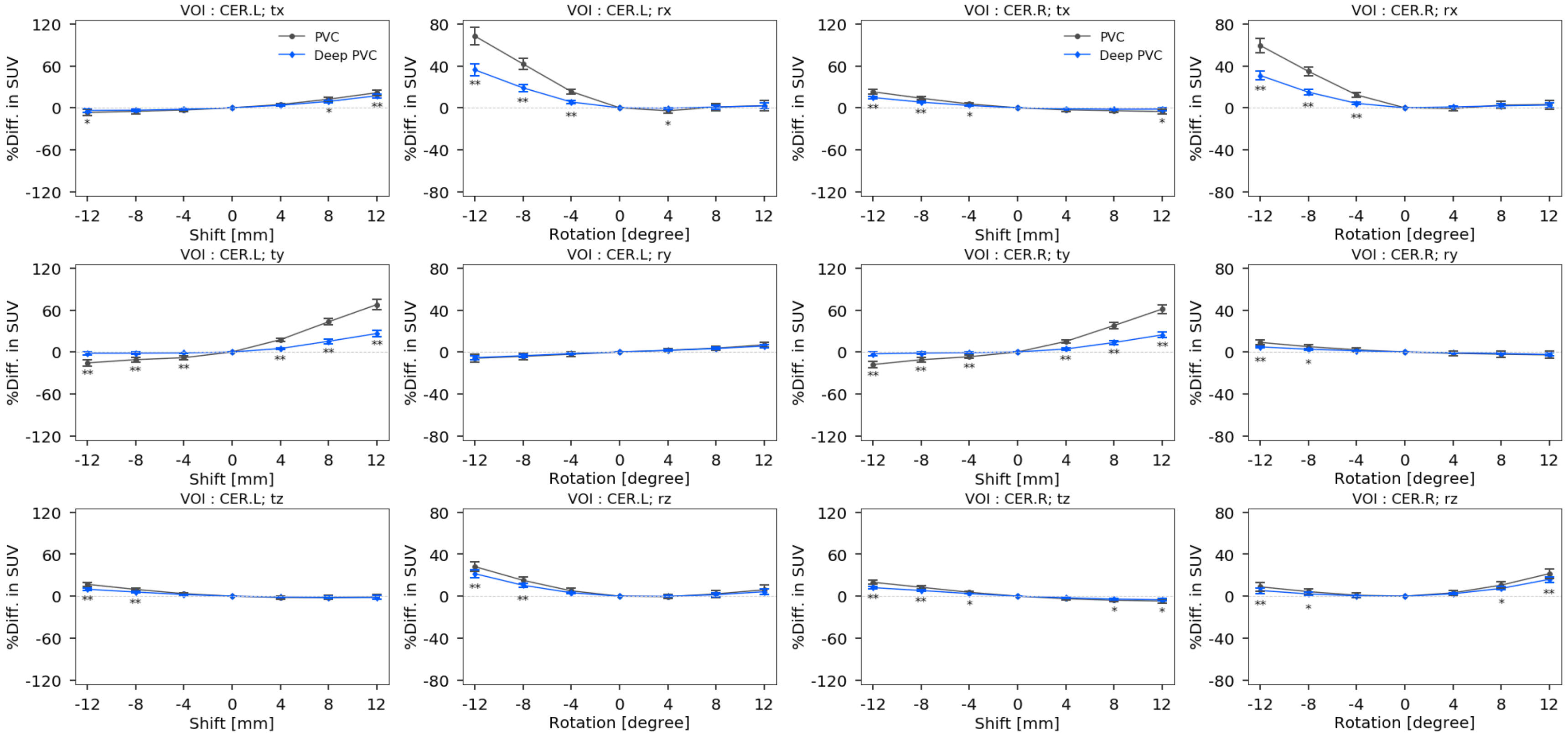

VOI : CER.L; tx
VOI : CER.L; rx
VOI : CER.R; tx
VOI : CER.R; rx
VOI : CER.L; ty
VOI : CER.L; ry
VOI : CER.R; ty
VOI : CER.R; ry
VOI : CER.L; tz
VOI : CER.L; rz
VOI : CER.R; tz
VOI : CER.R; rz
PVC
Deep PVC
%Diff. in SUV
Shift [mm]
Rotation [degree]

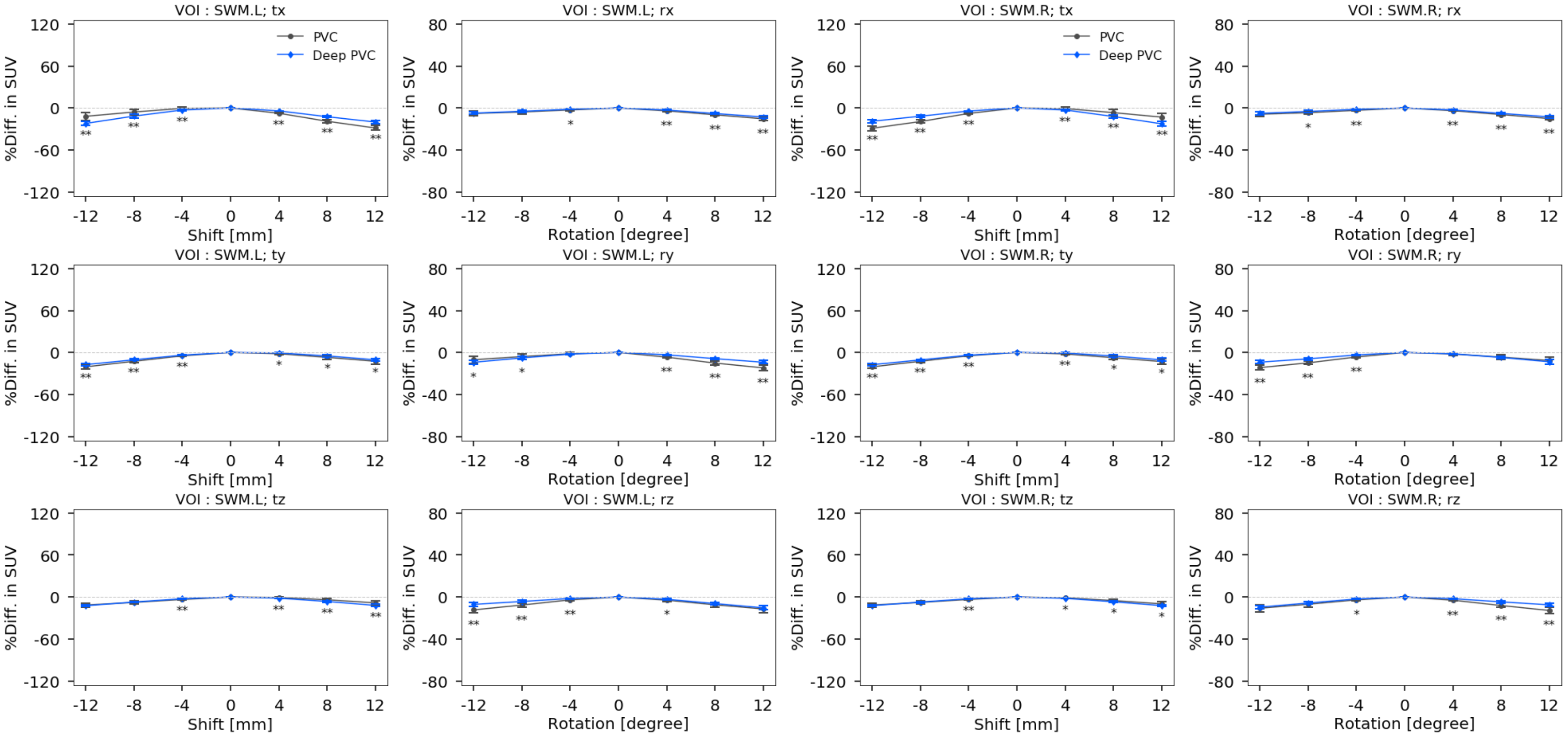
VOI : SWM.L; tx
VOI : SWM.L; rx
VOI : SWM.R; tx
VOI : SWM.R; rx
VOI : SWM.L; ty
VOI : SWM.L; ry
VOI : SWM.R; ty
VOI : SWM.R; ry
VOI : SWM.L; tz
VOI : SWM.L; rz
VOI : SWM.R; tz
VOI : SWM.R; rz
PVC
Deep PVC
%Diff. in SUV
Shift [mm]
Rotation [degree]
